# Block mapping and dual-matrix-based watermarking for image authentication with self-recovery capability

**DOI:** 10.1371/journal.pone.0297632

**Published:** 2024-02-02

**Authors:** Xuejing Li, Qiancheng Chen, Runfu Chu, Wei Wang

**Affiliations:** 1 Computer and soft Engineer Department, Anhui Institute of Information Technology, Wuhu, Anhui, China; 2 Shanghai DataSeed Information Technology, Shanghai, China; Kocaeli University, TURKEY

## Abstract

Numerous image authentication techniques have been devised to address the potential security issue of malicious tampering with image content since digital images can be easily duplicated, modified, transformed and diffused via the Internet transmission. However, the existing works still remain many shortcomings in terms of the recovery incapability and detection accuracy with extensive tampering. To improve the performance of tamper detection and image recovery, we present a block mapping and dual-matrix-based watermarking scheme for image authentication with self-recovery capability in this paper. The to-be-embedded watermark information is composed of the authentication data and recovery data. The Authentication Feature Composition Calculation algorithm is proposed to generate the authentication data for image tamper detection and localization. Furthermore, the recovery data for tampered region recovery is comprised of self-recovery bits and mapped-recovery bits. The Set Partition in Hierarchical Trees encoding algorithm is applied to obtain the self-recovery bits, whereas the Rehashing Model-based Block Mapping algorithm is proposed to obtain the mapped-recovery bits for retrieving the damaged codes caused by tampering. Subsequently, the watermark information is embedded into the original image as digital watermarking with the guidance of a dual-matrix. The experimental results demonstrate that comparing with other state-of-the-art works, our proposed scheme not only improves the performance in recovery, but also extends the limitation of tampering rate up to 90%. Furthermore, it obtains a desirable image quality above 40 dB, large watermark payload up to 3.169 bpp, and the effective resistance to malicious attack, such as copy-move and collage attacks.

## I. Introduction

The transmission of multimedia has become exceedingly convenient with the advent of networks and digital technology. As a result, digital images have been served as the crucial multimedia tool for information acquisition and sharing. Nevertheless, the security of digital multimedia is of great concern since these transmitted media can be easily duplicated, transformed, modified and diffused via the Internet. In this context, the integrity authentication technique based on multimedia content has aroused extensive attention over the past decades, especially for digital images [1]. To verify the authenticity of received media and locate the tampered regions, two research orientations have been proposed in passive [2] and active ways [3]. The passive authentication mechanism detects the image according to the post-processing traces, such as unnatural tampered boundaries [4], strong contrast difference [5], and so on. One common method is regarding the hash result of original image as the authentication information; thus, the legal user can declare the received image as unaltered if the hash result is identical with the one transmitted from original image [6–8]. Obviously, the implementation of hash-based image integrity verification is complicated since it requires a secure communication channel, which must be reused for each image transmission. Although these passive authentication mechanisms are effective in tamper detection and dispensed with additional data, they are not able to recover the tampered regions. In this light, the digital watermarking-based active image authentication mechanism is a more applicable scheme for image authentication owing to its easier implementation, capability of tampered region localization and self-recovery.

Generally, digital watermarking-based schemes for image authentication can be further divided into the categories of semi-fragile watermarking and complete fragile watermarking [9]. To be specific, the semi-fragile watermarking-based schemes [10, 11] are robust to certain attacks and can distinguish the genuine malicious tampering from ordinary signal processing. Whereas, the complete fragile watermarking-based schemes are sensitive to all tampering operations and can detect any modifications in the original image [12]. It is obviously that the complete fragile watermarking is more appropriate for the accurate image integrity authentication. Note that the general spatial domain-based image data hiding scheme cannot resist any modifications and it is pertain to the complete fragile watermarking. The authentication information is embedded into the original image to generate the watermarked image through the spatial domain-based data hiding schemes [13–15]; in this way, the modified pixel values will not be extracted correctly so as to achieve the purpose of accurate tamper detection. Correspondingly, we also choose the spatial domain-based image data hiding scheme to generate the complete fragile watermarking for the accurate image integrity authentication.

In recent years, the great challenge of watermarking-based image authentication techniques is the recovery incapability with respect to the extensive tampering. Furthermore, it is also confronted with the problem of improving the image visual quality and security. To address these problems, we propose a block mapping and dual-matrix-based digital watermarking scheme for image authentication with self-recovery capability. Considering that the metrics used to evaluate the performance of watermarking-based image authentication schemes include tamper detection accuracy, self-recovery capacity, watermark payload, image quality and security. The last three criteria are principally determined by the definite data hiding algorithms utilized in the construction of fragile watermarking. Since our purpose is to design complete fragile watermarking for the accurate image tamper detection and localization, numerous data hiding algorithms based on the spatial domain [16–18] can be taken into consideration to form a watermarked image with better performance. The classic least-significant-bit (LSB) substitution scheme [16] for data hiding was first presented by Bender et al., which directly replaces the LSBs of every pixel with binary stream. Some state-of-the-art image authentication techniques [13, 19, 20] also choose LSB to hide watermark information in the original image. Although LSB substitution mechanism is simple and efficient to implement, the quality of watermarked images drops sharply with the increment of embedding payload, and the concealed information can easily be detected via some uncomplicated statistical analysis attacks such as RS detection [21]. Therefore, we devise a novel data hiding algorithm with a large payload of 3 bpp and visual quality above 40 dB, as detailed in Section IV.

In this paper, the watermark information is composed of the authentication data for tampered region localization and the recovery data for image content self-recovery. Therefore, the Authentication Feature Composition Calculation (AFCC) algorithm is proposed to generate the authentication data, whereas the Set Partition in Hierarchical Trees (SPIHT) algorithm and the proposed block mapping algorithm are applied to generate the recovery data that contains self-recovery bits and mapped-recovery bits. The key contributions can be summarized as follows:

1. The AFCC algorithm is proposed to obtain authentication bits, including the parity check bits and hash indicator table (HIT) feature bits.
2. To generate the parity check bits, we design the Block-based DWT Parity Check (BDPC) algorithm that decomposes every block into diverse levels according to the image block size and then check the parity of corresponding DWT coefficients.
3. The Rehashing Model-based Block Mapping (RMBM) algorithm is proposed to generate the HIT feature bits and then combined with SPIHT encoding algorithm to generate the mapped-recovery bits.
4. To improve the visual quality and security of watermarked image, we devise the Dual-Matrix- based Data Hiding (DMDH) algorithm to embed watermark information into the original image.

The experimental results demonstrate that our proposed scheme outperforms the state-of-the-art works with respect to the accuracy of tampered region localization and image self-recovery performance, while obtaining a satisfactory watermarked image visual quality and the effective resistance to malicious attack.

The remaining parts of this paper are organized as follows. Section II introduces some related works in recent years. Then, Section III gives an abbreviated review of the rehashing model-based perfect hash function and SPIHT encoding algorithm. Section IV describes the proposed data hiding algorithm while Section V elaborates the proposed scheme for image tamper detection and self-recovery. The specific experimental results, the conclusions and further works will be discussed in Section VI and Section VII, respectively.

## II. Related work

In recent years, some state-of-the-art schemes in image tamper detection and self-recovery have been proposed. Lin et al. [15] proposed the block-based watermarking strategy, where one image block conceals the authentication data of itself and the recovery data of another image block, in order to locate the tampered regions more exactly. Inspired by this, some other block-based digital watermarking schemes for image authentication [19, 20, 22–24] have been created to improve the tamper detection performance. Lee and Lin [22] employed a dual watermark to provide the second opportunity for image tampered region recovery when the available information for content self-recovery was damaged for the first time. Thus, under 90% tampering rate, the quality of recovered image reconstructed by [22] is approximately 20 dB. Sarreshtedari et al. [19] introduced the Set Partitioning In Hierarchical Trees algorithm to engender the recovery bits and employed the hash function to obtain authentication bits. Furthermore, an erasure decoder with Reed-Solomon channel codes was conducted to retrieve the source encoded outputs. Under this premise, it can be made suitable for diverse intentions by adjusting the main parameters and efficiently recover the tampered image under 33% modification without any noticeable distortion. However, [19] can’t resist the copy- move attack since its authentication bits were constructed by independent image blocks. In 2019, Haghighi et al. [23] designed a novel fragile watermarking method with high tamper detection accuracy, especially for the extensive modifications. It constructed four compact digests to provide four opportunities for the tampered region recovery, and employed Mirror-aside and Partner-block to further improve the image recovery performance. Although the watermarked image quality reached up to 46 dB on average, the embedded watermark information can be easily damaged under some malicious tampering attacks. Prasad et al. [24] devised a secure fragile watermarking scheme for image authentication in 2020. The watermark bits are obtained from the most-significant-bits (MSB) of each pixel by Hamming code, and encrypted with secret binary bits generated by the Logistic map. However, the embedded bits with only authentication code will cause the failure of image recovery when it faced with tampering attack. Subsequently, Liu et al. [20] proposed an adaptive scheme for image tamper detection and content recovery, where the pixel- based diagonal mapping is used to obtain the recovery data of tampered regions. But the quantitative location information of block is not accurate as authentication data, the corresponding tamper detection accuracy needs to be improved. Singh et al. [25] presented an improved tamper detection and self-recovery scheme, and the restoration bits were generated by encoding the content from two levels. Hence, the tampered image with half content modification can still be restored, but it may lose the recovery ability when faced with certain image processing operations. In [26], Qin et al. introduced a novel image tamper detection technique based on pixel-wise fragile watermarking, where the reference bits were derived from each overlapping block and then embedded into the original image. Sreenivas et al. [27] proposed to generate the authentication bits from 2 × 2 image block, and one set of recovery bits was concealed in another randomly chosen block. However, the security of this scheme needs to be improved since it has no resistance to certain common attacks, such as copy-move. Then, Sahu et al. [28] proposed a novel dual image-based reversible fragile watermarking scheme, which embeds two secret bits in each host image pixel using a pixel readjustment strategy to obtain dual watermarked images. Although it can accurately detect and locate the tampering regions from an image, the dual watermarked images need more secret bits to obtain the aforementioned performance. Barani et al. [29] proposed a new grayscale image authentication technique in the integer wavelet transform domain, which used a 3D quantum map to prevent security problem for the algorithm. In order to avoid unauthorized access to multimedia content in real-time data transmission, Sahu [30] proposed a logistic map based fragile watermarking technique to efficiently detect and localize the tampered regions from the watermarked image. This scheme takes advantage of the sensitivity property of the logistic map to generate watermark bits, which are embedded in the rightmost LSBs by performing the logical XOR operation between the first intermediate significant bits and the watermark bits. In [31], logistic-map based fragile image watermarking scheme for tamper detection and localization is proposed, which is blind and the watermark bits are generated using the chaotic system based logistic-map at both the sender and receiving end. Therefore, the quality of the watermarked image is quite superior to that of the other schemes and the outstanding results are achieved with respect to the tamper detection and localization ability. However, this scheme has shortcomings in terms of the recovery incapability with tampered regions.

Generally, the existing works still remain many shortcomings in terms of the recovery incapability and detection accuracy with extensive tampering. Furthermore, it is also confronted with the problem of improving the watermarked image visual quality and security. To settle these problems, we propose a block mapping and dual-matrix-based watermarking scheme for image authentication with self-recovery capability in this paper.

## III. Preliminaries

To facilitate the comprehension of our proposed scheme, the fundamental knowledge of rehashing model-based perfect hash function and SPIHT encoding algorithm are separately discussed as follows.

### A. Rehashing model

Hashing is a function of mapping the key space into the address space, which is regarded as an efficient approach to organize and retrieve information. Note that if a hash function can one-to-one map from the series of keys in the key space to the address space, it can effectively avoid the key collision problem and is denoted as the perfect hash function. In this paper, we employ rehashing model [32] to design the perfect hash function.

Let *n* distinct keys *K*_1_, *K*_2_,⋯,*K*_*n*_ in the key space be respectively mapped to *m* entries *A*_1_, *A*_2_,⋯,*A*_*m*_ in the address space via a single hash function *h*_*k*_, which is randomly chosen in a set of mapping functions *F*_*n*×*m*_. Accordingly, the chance of selecting *h*_*k*_ as a perfect hash function is generally quite small; that is, in most instances, numerous collisions would occur in the address space with a single random hash function. To theoretically calculate the probability *P*_*i*_(*m*,*n*) that denotes the random hash function has *i* (0 ≤ *i* ≤ *min*(*m*,*n*)) entries in the address space with only one key mapped to them, we define the following (1).

$$P_{i}(m,n)=\frac{e_{i}(m,n)}{m^{n}},$$ (1)

where *e*_*i*_(*m*,*n*) can be further computed by (2).


$$e_{i}(m,n)=n!\left(\begin{array}{c} m\\ i \end{array}\right)\sum_{r=0}^{n-i}{\left(-1\right)}^{r}\left(\begin{array}{c} m-i\\ r \end{array}\right)\frac{{\left(m-r-i\right)}^{n-r-i}}{(n-r-i)!}$$ (2)

The expected values of singleton *i* for the probability distribution *P*_*i*_(*m*,*n*) are 3.87, 7.55, and 11.22; additionally, the corresponding probabilities are 1.6700%, 0.0205%, and 0.0003% in the case of *i* ≥ 0.8 × *n* and *m* = *n* = 10, 20, 30. However, the rehashing model constituted by seven random hash functions can eliminate numerous collisions, and HIT stores the numerical order of selected hash functions that corresponds to the entry in address space. Denote $P_{i}^{k}(m,n)$ as the probability of having *i*(0 ≤ *i* ≤ *min*(*m*,*n*)) singletons in the address space with the rehashing model of *h*_1_, *h*_2_,⋯,*h*_*k*_ functions. Thus, the concrete definition is calculated by (3).

$$P_{i}^{k}(m,n)=\sum_{r=0}^{i}P_{r}^{k-1}(m,n)\times Q_{i-r}(m,n,r),$$ (3)

where $P_{i}^{1}(m,n)=P_{i}(m,n)$ and *Q*_*i*_(*m*, *n*, *j*) is described as the following (4).


$$Q_{i}(m,n,j)={\left(\frac{j}{m}\right)}^{n-j}\sum_{r=0}^{n-j}\left(\begin{array}{c} n-j\\ r \end{array}\right)\frac{e_{i}(r,m-j)}{j^{r}}$$ (4)

In this case, the expected values of singleton *i* for $P_{i}^{7}(m,n)$ are separately 8.80, 17.46, and 26.07 while the corresponding probabilities are 96.41%, 97.17%, and 97.84% for *i* ≥ 0.8 × *n* and *n* = *m* = 10, 20, 30, respectively. The above result verify that a rehashing model composed of only seven random hash functions can eliminate many collisions effectively. According to it, we propose the Rehashing Model-based Block Mapping (RMBM) algorithm to generate HIT feature bits for the sake of image tamper detection and localization.

### B. SPIHT encoding algorithm

Set partitioning in hierarchical trees (SPIHT) encoding [33] is an embedded compression algorithm widely applied in the image compression, which can transmit the output bit stream of the original image at the desired rate and reconstruct the decoded image with a high visual quality. It is mentioned that the larger output rate exploited, the higher visual quality of reconstructed image can be obtained. Hence, a sophisticated sorting algorithm is required to efficiently sort the coefficients after wavelet transform. The SPIHT algorithm applies the self- similarities among the diverse sub-bands of wavelet transform to encode, and these similarities can be found through the spatial-orientations tree of image wavelet decomposition. To be specific, it sorts the rounded multi-resolution wavelet transform coefficients according to their magnitudes and transmits them based on significant bit order.

Our algorithm aims to encode the 8 bits per pixel (bpp) original grayscale image and truncate the SPIHT output stream at the rate of 1 bpp. Note that under the malicious tampering with image content, the watermark information concealed in the tampered regions is irreversibly damaged. Since the SPIHT encoding algorithm sorts wavelet transform coefficients in the significant bit order, the damage of coefficients will impact the quality of recovered image and the capability of decoding. Furthermore, the image authentication techniques will lose recovery capability in the cases of extensive tampering. To address this problem, we propose to employ the recovery data composed of the self-recovery bits and mapped-recovery bits, which provides a guarantee of recovered image quality. Here, the SPIHT encoding algorithm is combined with the proposed RMBM algorithm to generate the recovery data for tampered region self-recovery.

## IV. Proposed watermark embedding and extraction

**Fig 1:**
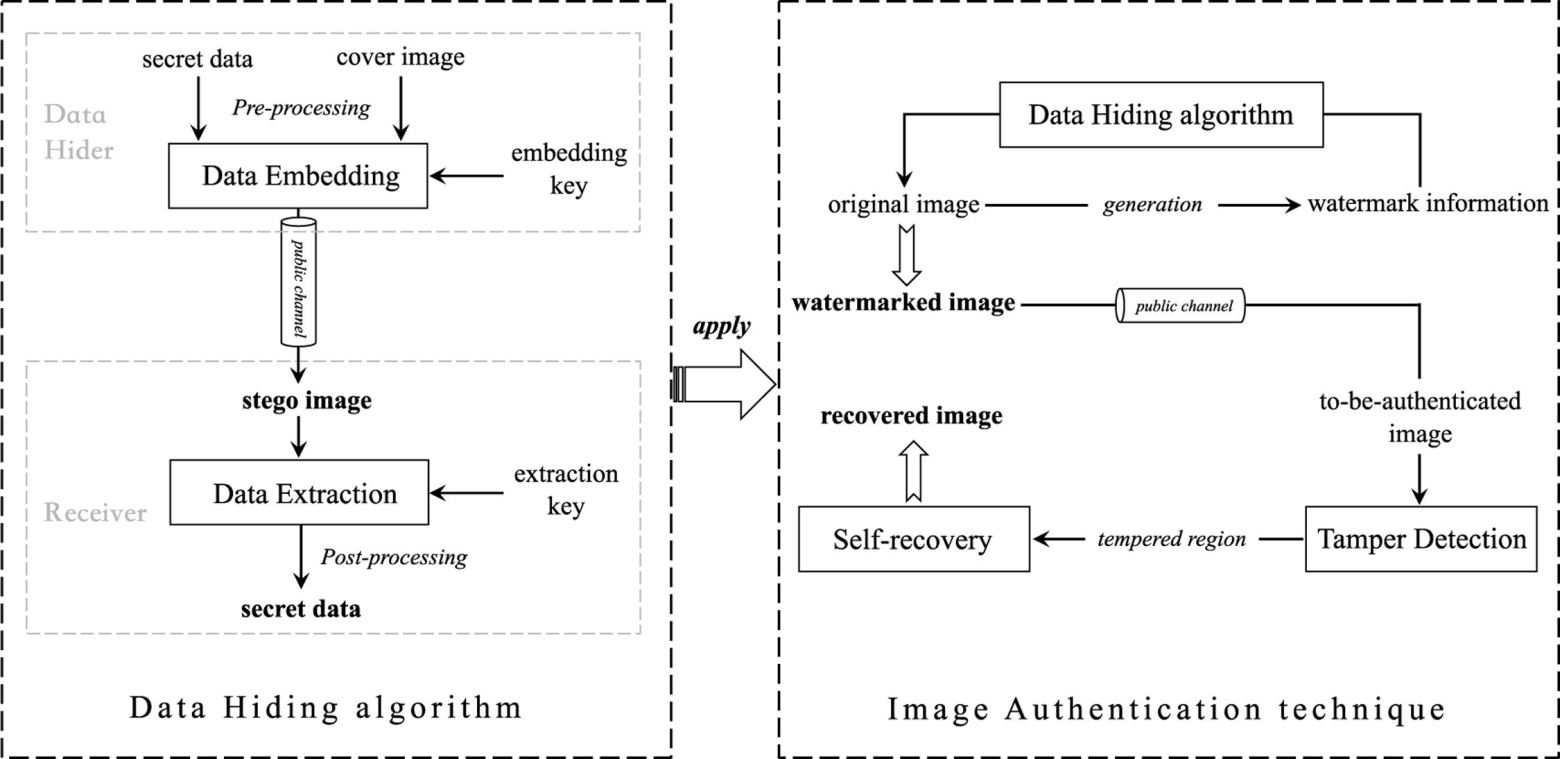
Schematic diagram of the proposed scheme.

In this paper, the watermark information composed of the authentication data and recovery data is embedded into the original image via dual-matrix, which can be further extracted for tamper detection and self-recovery. The schematic diagram of our proposed scheme is presented in Fig 1, it is generally divided into two phases: 1) Data hiding algorithm; 2) Image authentication technique.

Specifically, in the implementation of data hiding algorithm, the data hider should initially pre-process the secret data and cover image, and then feed them to the data embedding module together with the embedding key to construct a stego image. After receiving the stego image through the public communication channel, the legal receiver, who has the extraction key, can exactly obtain the secret data through the data extraction and post-processing. Since the modification direction in many data hiding algorithms [16–18] is limited, the payload of to-be-embedded watermark information cannot satisfy our demand. In this context, a novel Dual-Matrix based Data Hiding (DMDH) algorithm discussed in this section is proposed for the watermark embedding and extraction. The procedure of constructing this dual-matrix, and the detailed embedding and extraction implementation are presented in subsections IV-A and IV-B. In the implementation of image authentication technique, the authentication data and recovery data are firstly generated from the original image, which are regarded as watermark information, and then embedded into itself via DMDH so as to construct the watermarked image. Subsequently, the tamper detection and recovery operation for tampered regions can be performed on the to-be-authenticated image that is received from the public communication channel.

### A. Construction of dual-matrix

**Fig 2:**
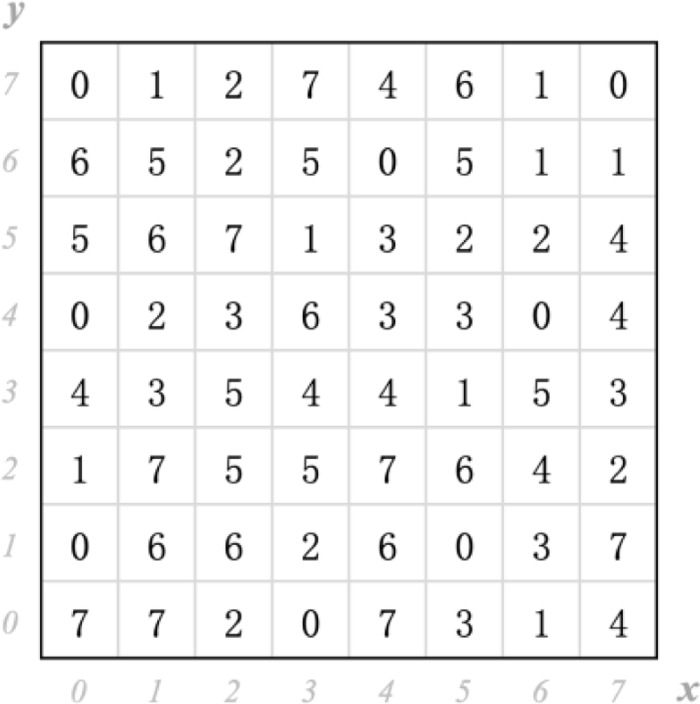
Demonstration of a puzzle *P* with the dual embedding property.

To satisfy the demand of large watermark payload in this paper, a novel DMDH algorithm is proposed, where both of the first-order and the second-order embedding space in dual- matrix DM is adequately exploited, and thus it can guides two 8-ary notational system secret digits to be simultaneously embedded into each cover pixel pair. Consequently, the devised *DM* is composed of several 8 × 8 puzzles *P*, and the selected puzzle must enclose all non-repetitive combinations of 8-ary digits. Obviously, many optional puzzles *P* fulfil the aforementioned condition, and an example is demonstrated in Fig 2.

This selected puzzle *P* has the dual embedding space; viz., the coordinate (*x*, *y*) of *P* in the first-order embedding space denotes *d*_1_ and the coordinate (*x* + 1 *mod* 8,*y*) of *P* in the second-order embedding space denotes *d*_2_. Furthermore, all groups of *d*_1_ and *d*_2_ can represent the diverse combinations in 8-ary digital format defined as (5).


$$\left\{\begin{array}{c} P(x,y)=d_{1}\\ P(x+1\;mod\;8,y)=d_{2} \end{array}\right.$$ (5)

**Fig 3:**
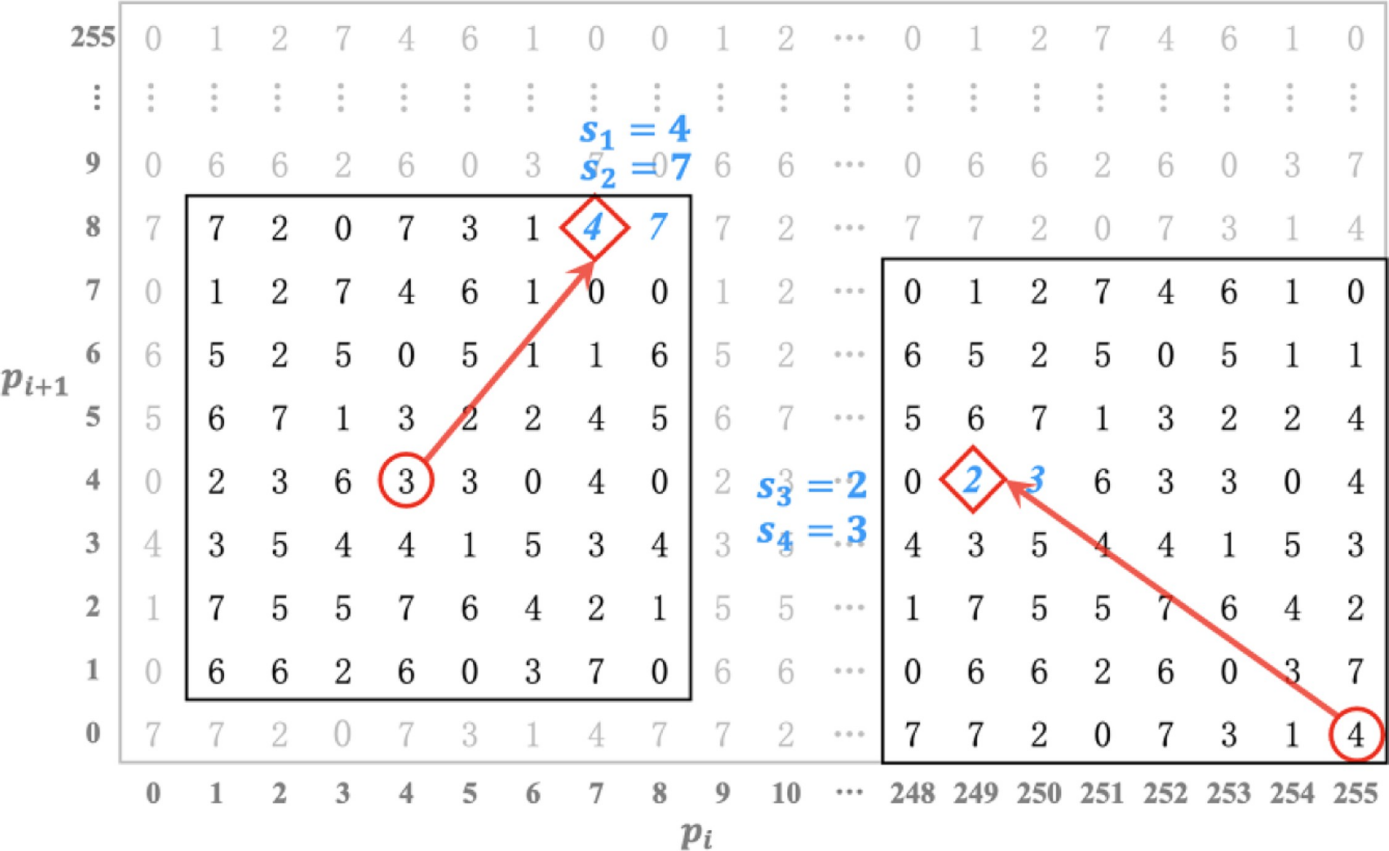
The selected 256 × 256 dual-matrix *DM*.

To construct *DM*, as presented in Fig 3, the 8 × 8 puzzle *P* is tiled repeatedly and then the formative matrix is truncated to a two-dimensional reference matrix with a size of 256 × 256.

Thus, the pixel pair (*p*_*i*_, *p*_*i*+1_) retrieved from original image *I* is located at the position *DM*(*p*_*i*_, *p*_*i*+1_), where the variables *p*_*i*_ and *p*_*i*+1_ separately imply the *p*_*i*_-th row and *p*_*i*+1_-th column of *DM*. Subsequently, the corresponding novel 8 × 8 sub-puzzle $P^{\prime }$ can be directly constructed with the center of *DM*(*p*_*i*_, *p*_*i*+1_) in the case that 3 ≤ *p*_*i*_,*p*_*i*+1_ ≤ 252; otherwise, it can be reconstructed along with the border line of *DM*. Besides, this novel established $P^{\prime }$ has the dual embedding property as well.

### B. Implementation of embedding and extraction

As is evident from the preceding analysis, the core idea of this proposed DMDH algorithm is to embed two base-8 digits into each pixel pair synchronously via referring to the selected dual-matrix *DM*. Assume that a binary stream *B* with *l* bits is the watermark information that contains all authentication data and recovery data *S* to be embedded; meanwhile, the original image *I* sized *h* × *w* is the host image to conceal these data, where the parameters *h* and *w* separately represent the height and width of *I*. The implementation of the embedding and extraction procedure is elaborated as follows.

1) *Data Embedding Procedure*: In the pre-processing, we scan the original image *I* in zig-zag order through rows into a one-dimensional pixel sequence and then divide all the image pixels into a series of the non-overlapping original pixel pairs (*p*_*i*_, *p*_*i*+1_), where *p*_*i*_ and *p*_*i*+1_ are the grayscale values of two adjacent pixels and the parameter *i* is sequentially chosen from {1,3,⋯,*h* × *w*– 1}. Additionally, the to-be-concealed binary stream *B* is split into several segments and then converted into 8-ary notational system digits, viz., *B* = (*b*_1_, *b*_2_,⋯,*b*_*l*_)_2_ = (*s*_1_, *s*_2_,⋯,*s*_*h*×*w*_)_8_ = *S*, where *l* = *h* × *w* × 3 and it represents the sum total of binary bits. Subsequently, the concrete data embedding can be proceeded with ***Algorithm I***.

***Algorithm I*** *Data Embedding Procedure*
**Input:** Original image *I* sized *h* × *w*, binary stream *B* and a selected 8 × 8 puzzle *P*
**Output:** Watermarked image *I_w_* sized *h* × *w*
1: Divide *I* into pixel pair sequence (*p_i_*, *p_i_*_+1_)
2: Convert *B* into an 8-ary digital stream *S*
3: Construct the 256 × 256 reference matrix *DM* according to *P*
4: **for** *i = 1:2: h × w—1* **do**
5:  **if** there left some secret data to be embedded **then**
6:  Scan pixel pair (*p_i_*, *p_i_*_+1_)
7:  Locate the coordinate (*p_i_*, *p_i_*_+1_) in *DM* at *DM*(*p_i_*, *p_i_*_+1_)
8:  Construct a novel sub-puzzle $P^{\prime }$ according to *DM*(*p_i_*, *p_i_*_+1_)
9:  Retrieve secret digits (*s_i_*, *s_i_*_+1_)_8_ from *S*
10:  Find $\left({p_{i}}^{\prime },{p_{i+1}}^{\prime }\right)$ in $P^{\prime }$ satisfying the (5)
11:  Replace (p_i_, p_i+1_) with $\left({p_{i}}^{\prime },{p_{i+1}}^{\prime }\right)$ in the original image
12:  **else** break
13:  **else if**
14: **end for**

An example is demonstrated as follows to facilitate the comprehension of this embedding procedure.

***Example 1***. Assume the non-overlapping pixel pairs in original image *I* are (4,4) and (255,0) while the binary stream to be embedded is *B* = (100111 010011)_2_, which can be converted into 8-ary digits and further split into two segments (47)_8_ and (23)_8_. The specific procedure is illustrated in Fig 5, where the red circles are the original localizations in the *DM* and the rhombuses that the solid arrows point to are the final modified pixel pairs. Some detailed descriptions are supplemented below.

(i). Embed the base-8 digits *s*_1_ = (4)_8_ and *s*_2_ = (7)_8_ into the first original pixel pair (4,4).

Locate the pixel pair (4,4) at coordinate *DM*(4,4) by referring to the dual-matrix *DM* and then establish a novel sub-puzzle with the center of it. Obviously, the value of *DM*(4,4) is not equal to *s*_1_; therefore, the elements in this sub-puzzle are fully searched to choose all qualified coordinates that fulfil $DM\left(p_{i}^{\prime },p_{i+1}^{\prime }\right)=s_{1}$. According to (5), the adjacent coordinates in this novel established sub-puzzle can be ascertained. In this light, the ultimate modified position (7,8) is determined since its subsequent value is equivalent to the 8-ary digit *s*_2_.

(ii). Embed the base-8 digits *s*_3_ = (2)_8_ and *s*_4_ = (3)_8_ into the second original pixel pair (255,0).

The definite localization of *DM*(255,0) is fixed according to the reference matrix; in addition, we further reconstruct a novel sub-puzzle with the center of *DM*(251,3). Consequently, the final modified pixel pair(249,4) in this sub-puzzle can be ascertained since *DM*(249,4) = *s*_3_, and its contiguous element *DM*(250,4) of it is also identical to the digit *s*_4_.

2) *Data Extraction Procedure*: Upon receiving the watermarked image *I*_*w*_ sized *h* × *w* and puzzle *P* employed in the previous embedding implementation, the legal user can precisely extract the concealed information *B* by means of ***Algorithm II***. The puzzle *P* is tiled repeatedly to construct the reference matrix *DM*, which is truncated to a size of 257 × 257, for facilitating the extraction operation. Therefore, the current pixel pair is located at $DM({p_{i}}^{\prime },{p_{i+1}}^{\prime })$. Accordingly, the first concealed base-8 digit *s*_*i*_ can be directly confirmed as per $DM\left({p_{i}}^{\prime },{p_{i+1}}^{\prime }\right),$ and the second digit *s*_*i*+1_ is continuously calculated as per its adjacent coordinate value about abscissa. Here, the two extracted 8-ary digits satisfy (6).


$$\left\{\begin{array}{c} s_{i}=DM\left({p_{i}}^{\prime },{p_{i+1}}^{\prime }\right)\\ s_{i+1}=DM\left({p_{i}}^{\prime }+1,{p_{i+1}}^{\prime }\right) \end{array}\;0\leq {p_{i}}^{\prime },{p_{i+1}}^{\prime }\leq 255\right.$$ (6)

***Algorithm II*** *Data Extraction Procedure*
**Input:** Watermarked image *I_w_* sized *h* × *w* and the selected puzzle *P*
**Output:** Binary stream *B*
1: Divide *I_w_* into pixel pair sequence $\left({p_{i}}^{\prime },{p_{i+1}}^{\prime }\right)$
2: Construct the 257 × 257 reference matrix *DM* according to *P*
3: **for** *i = 1:2: h × w—1* **do**
4:  **if** there left some secret data to be extracted **then**
5:  Scan pixel pair $\left({p_{i}}^{\prime },{p_{i+1}}^{\prime }\right)$ from *I_w_*
6:  Locate the coordinate $\left(p_{i}^{\prime },p_{i+1}^{\prime }\right)$ in *DM* at $DM\left({p_{i}}^{\prime },{p_{i+1}}^{\prime }\right)$
7:  Calculate secret digits (*s_i_,s_i+1_*)_8_ according to the (6)
8:  **else** break
9:  **else if**
10: **end for**
11: Convert *S* into the binary stream *B*

Similarly, provide a demonstration to exemplify the extraction procedure.

***Example 2***. Assume that stego pixel pair $\left({p_{i}}^{\prime },{p_{i+1}}^{\prime }\right)$ in the received watermark image *I*_*w*_ is (7,8), so the corresponding subsequent coordinate of *DM*(7,8) can be computed as *DM*(8,8). According to the previous (6), two base-8 hidden digits are extracted as (47)_8_ and then further converted into binary stream *B* = (100111)_2_.

## V. Proposed image authentication scheme

To improve the tamper detection accuracy and image self- recovery performance, we devise an efficient block mapping and dual-matrix-based complete fragile watermarking scheme for image authentication with self-recovery capability in this paper. The core regulation of our proposed scheme is taking non-overlapping 2 × 2 pixels as an image block and then embedding the watermark information into the corresponding image block via the DMDH algorithm. Both the authentication data for tampered region localization and the recovery data for image content recovery will constitute watermark information. Concretely, the phase of this scheme can be implemented as follows: The design of the rehashing model is described in subsection V-A while the implementation of watermarked image construction, image authentication and self-recovery is illustrated in subsections V-B and V-C. For simplicity, the main symbols employed in describing our proposed scheme and their corresponding definitions are listed in Table 1.

**Table 1:** Main symbols employed in our scheme and their corresponding definitions.

Symbols	Definitions
*I*	Original image
*I*_*w*_	Watermarked image
$I^{\prime }$	Received image
*I*_*t*_	Tamper detection image
*I*_*r*_	Recovered image
*h*, *w*	The height and width of image
*B*_*u*_	Image block with *u*-th 2 × 2 pixels
*S*	Watermark information
HIT	Hash indicator table
HAT	Hash address table
FAT	Flag address table
IHAT	Inverse hash address table
TDL	Tamper detection location
RL	Recovery location
SRL	Self-recovery location
MRL	Mapped-recovery location
*DM*	Dual-matrix

### A. Design of rehashing model

For a natural image, many different pixels have identical grayscale pixel values. They would result in the same hashing values in address space, which cannot be discriminated from the other image pixels; therefore, the value of a pixel is not appropriate for a hashing key. Note that the localizations of each pixel in any image are disparate, which can serve as the keys of hash function and be further employed in the proposed Rehashing Model based Block Mapping (RMBM) algorithm. To be specific, the seed *s* of a random number generator (RNG) is utilized to engender seven random integers *S*_1_, *S*_2_,⋯,*S*_7_ and then applied to RNG for constructing the corresponding hash functions *h*_1_, *h*_2_,⋯,*h*_7_, respectively. Herein, the size of key space is *h* × *w*/4 while the hash value of seven functions *h*_1_, *h*_2_,⋯,*h*_7_ ranges from 1 to *h* × *w*/4; in a nutshell, the hash functions used here satisfy the following (7). Among them, *h*_*k*_(*u*) denotes the address value of key space *u* (i.e., the image block *B*_*u*_’s serial number), which is calculated by random hash function *h*_*k*_.


$$1\leq h_{k}(u)\leq \frac{h\times w}{4},1\leq k\leq 7\;\text{and}\;1\leq u\leq \;\frac{h\times w}{4}$$ (7)

**Fig 4:**
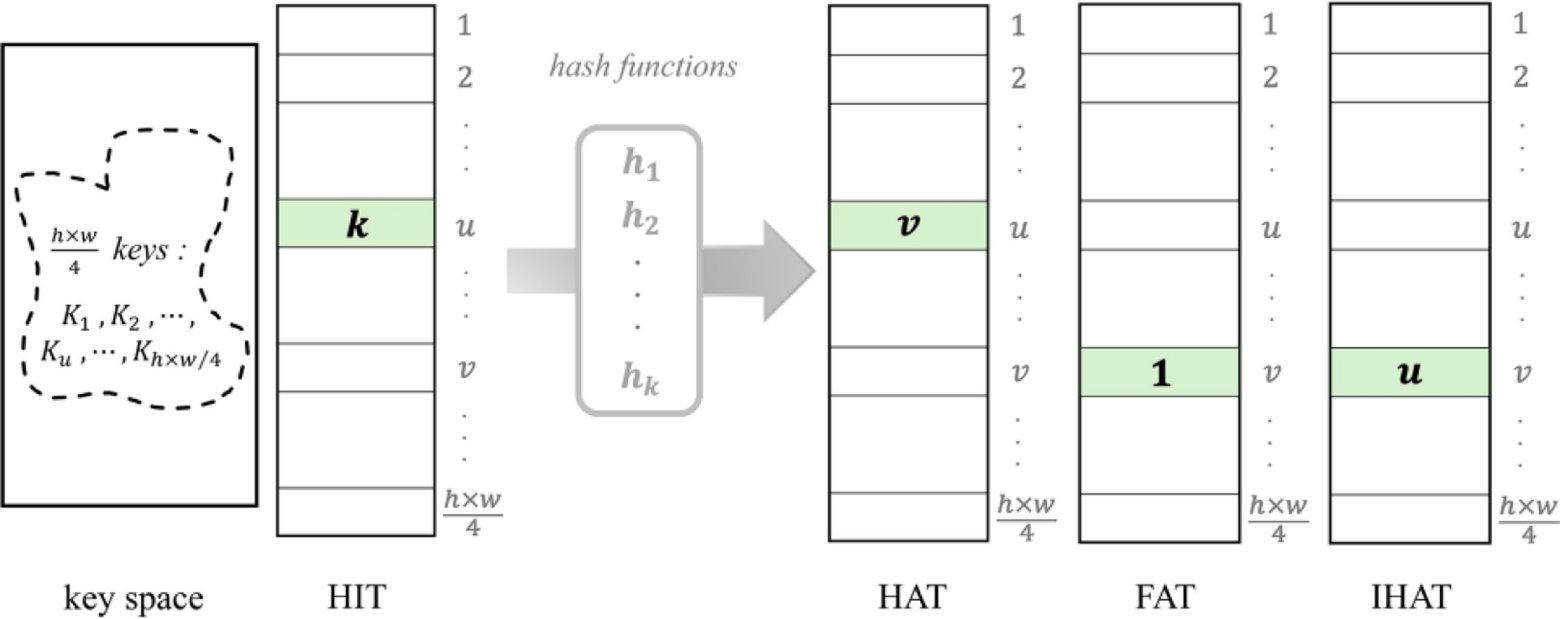
Design of the HIT, HAT, FAT and IHAT.

Except for the HIT, the RMBM algorithm employed in our proposed scheme should additionally design the hash address table (HAT), flag address table (FAT) and inverse hash address table (IHAT) to be suitable for image tamper detection and recovery. HIT is used to indicate the numerical order of selected hash function, while HAT stores the corresponding hash results in address space. FAT is designed for expediting the operational efficiency, which can indicate whether the address unit in HAT has been occupied. Furthermore, the IHAT of original image is constructed to facilitate the image recovery process. Fig 4 shows an instance for the arrangement of these tables. Actually, only HIT is absolutely essential for the subsequent image authentication operation since we can recompute the corresponding HAT and IHAT according to *s* and HIT. The implementation is elaborated as ***Algorithm III***.

***Algorithm III*** *Rehashing Model based Block Mapping algorithm*
**Input:** A random number seed *s* and the size of original image *I*:*h* × *w*
**Output:** HIT, HAT, IHAT and FAT corresponding to the original image *I*
1: Construct random hash functions *h_1_,h_2_,⋯,h_7_* according to *s*
2: HIT[*1:h×w/4] = 0*
3: HAT[*1:h×w/4] = 0*
4: IHAT[*1:h×w/4] = 0*
5: FAT[*1:h×w/4] = 0*
6: **for** *u = 1:h×w/4* **do**
7:  initialize indicator *k = 1*
8:  **while** *k ≤ 7* do
9:   **if** *FAT(h_k_(u)) = 0* **then**
10:    *FAT(h_k_(u)) = 1*
11:   HIT*(u) = k*
12:   HAT*(u) = h_k_(u)*
13:   **else**
14:   *k = k + 1*
15:   **end if**
16:  **end while**
17: **end for**
18: **for** *u = 1:h×w/4* **do**
19:  if *HIT*(u) = 0 **then**
20:   find unoccupied address *v* in sequence
21:   FAT*(v) = 1*
22:   HAT*(u) = v*
23:  **end if**
24: **end for**
25: **for** *u = 1:h×w/4* **do**
26:   IHAT(*HAT(u)*) = *u*
27: **end for**

### B. Construction of watermarked image

**Fig 5:**
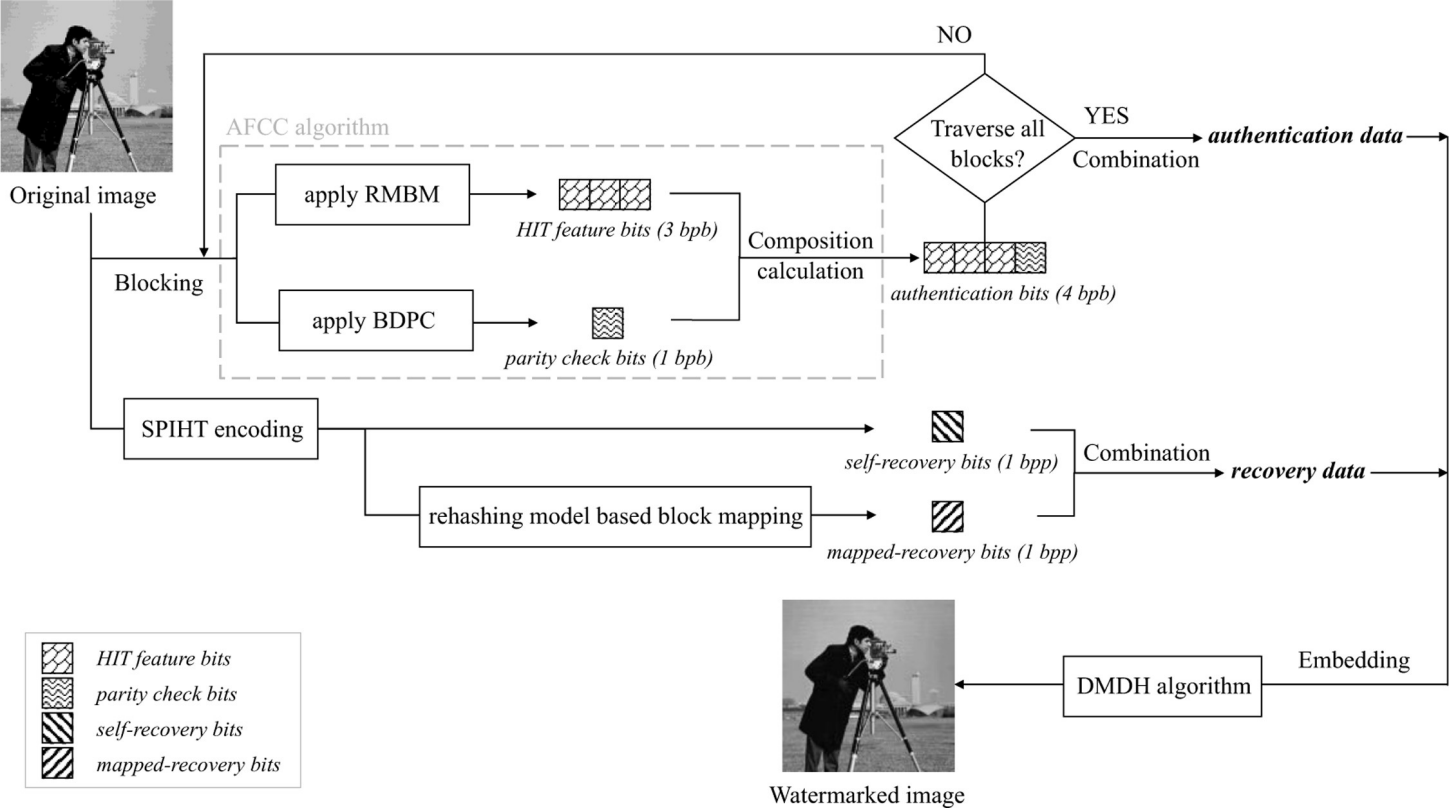
Illustration of constructing the watermarked image for authentication.

Fig 5 illustrates the flowchart of constructing watermarked image for authentication. The original image *I* with a size of *h* × *w* is firstly divided into non-overlapping 2 × 2 blocks and it fulfils *h*, *w mod* 2 = 0; thus, the sum total of segmented image blocks *B*_*u*_ is *h* × *w/4*. Subsequently, the proposed Authentication Feature Composition Calculation (AFCC) algorithm is employed to obtain the authentication bits *A*_*b*_ with 4 bits per block (bpb), which is composed of HIT feature bits *A*_*b*_*h*_ and parity check bits *A*_*b*_*p*_. The designed RMBM algorithm executed as ***Algorithm III*** is applied to generate *A*_*b*_*h*_ with 3 bpb. The Block-based DWT Parity Check (BDPC) algorithm, which decomposes every block into diverse levels according to the image block size and checks the parity of the corresponding DWT coefficients, is proposed to generate *A*_*b*_*p*_ with 1 bpb. By each DWT decomposition, four sub-band LL, LH, HL, and HH are constructed, in which the LL sub-band contains the approximation information of image blocks while the remaining three sub-bands contain the detailed information. Hence, the LL sub-band is chosen for the next decomposition process, and the number of iterations is determined by the image block size. After the multi-level decomposition, only one coefficient will be retained in the sub-band LL, and *A*_*b*_*p*_ corresponds to each image block can be calculated according to the parity of it. Furthermore, the authentication data *D*_*a*_ can be constructed by combining all the *A*_*b*_ of traversed blocks.

Simultaneously, the SPIHT encoding algorithm is used to generate the self-recovery bits *R*_*s*_ and then combined with the proposed RMBM algorithm to generate the mapped-recovery bits *R*_*m*_ that can retrieve the lost recovery data caused by tampering and provide a guarantee of recovered image visual quality. Both *R*_*s*_ and *R*_*m*_ can be further used to construct the recovery data *D*_*r*_. For the *D*_*r*_ of *B*_*u*_, its *R*_*s*_ corresponds to the image block *B*_*u*_, while *R*_*m*_ corresponds to the image block *B*_*IHAT*(*u*)_. Ultimately, *D*_*a*_ for image tamper detection and *D*_*r*_ for image self-recovery are regarded as watermark information *S*, which is embedded into the corresponding image block via DMDH algorithm. Therefore, the watermarked image *I*_*w*_ for authentication can be constructed as ***Algorithm I***.

### *C*. Image authentication and self-recovery

**Fig 6:**
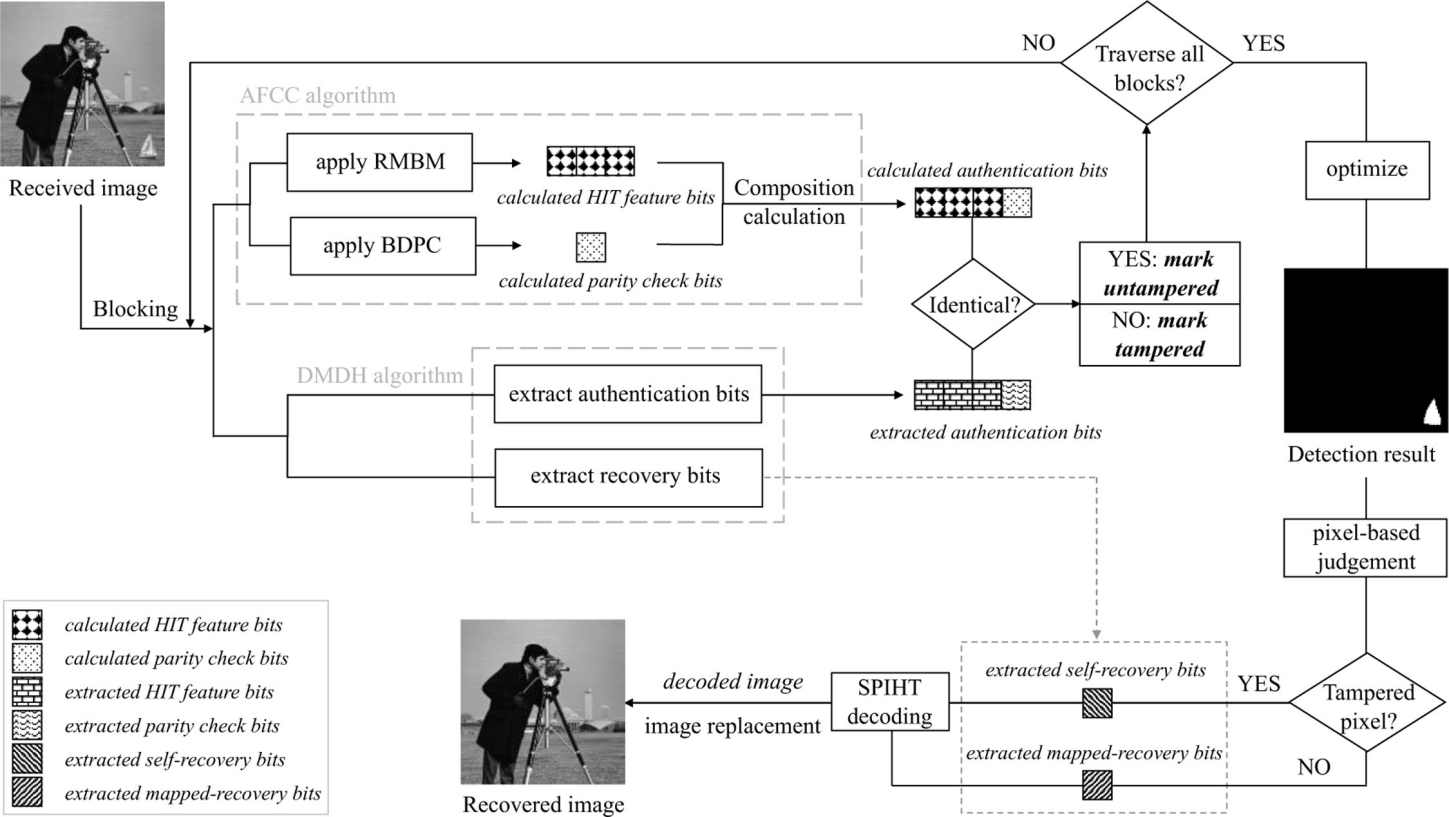
Illustration of authenticating and recovering the received image.

After constructing the watermarked image embedded the authentication data and recovery data, the process of image integrity authentication and self-recovery is discussed in this subsection. For the received image $I^{\prime }$, our proposed scheme can detect its tampered regions and further recover it to a desirable perceptual quality. Fig 6 illustrates the flowchart of authenticating and recovering the received image. Moreover, some detailed descriptions are supplemented below.

#### 1) Tamper detection and localization

Upon obtaining the image $I^{\prime }$ to be authenticated, the legal receiver who possesses the shared information of a random number seed *s* and puzzle *P* can authenticate the integrity of received image, i.e., verify whether the content of $I^{\prime }$ has been tampered with and then locate the tampered regions. In this light, a table of tamper detection location (TDL) is established to locate the tampered regions and facilitate the subsequent image recovery phase. The detailed steps are implemented as below.

***Input*:** Received image $I^{\prime }$ sized *h* × *w*, a random number seed *s* and the selected 8 × 8 puzzle *P*.

***Output*:** Tamper detection location TDL ranging from 1 to *h* × *w/4* and the tamper detection result *I*_*t*_.

#### Steps of the tamper detection and localization

Step 1: (*Pre-processing*)

Segment and reorganize the received image $I^{\prime }$ with zig-zag order through rows into an array of the non-overlapping 2 × 2 image blocks *B*_*u*_ where *u* ∈{1,2,⋯,*h* × *w*/4}, and initialize the contents of TDL table to 0. Then, the RMBM as ***Algorithm III*** and the BDPC algorithm are separately applied to calculate the HIT feature bits and parity check bits, which compose the calculated authentication bits *A*_*b*__*C*.

Step 2: (*Authentication*)

Retrieve the image block *B*_*u*_ sequentially and extract the corresponding authentication bits *A*_*b*__*E* as ***Algorithm II***. By comparing *A*_*b*__*C* and *A*_*b*__*E*, we can determine whether image block *B*_*u*_ has been tampered with. If these values are same, no modification of *TDL*(*u*) is required; otherwise, set *TDL*(*u*) = 1, denoting that this image block *B*_*u*_ has been tampered with.

Step 3: (*Loop judgment*)

Set *u* = *u* + 1, then proceed with Step 2 repeatedly until the value of *u* is equivalent to *h* × *w*/4. Ultimately, the whole TDL table for the received image $I^{\prime }$ is obtained.

Step 4: (*Visualization*)

Employ (8) to visualize the tampered image blocks in $I^{\prime }$.

$$I_{t}(x,y)=TDL(u)\;\;s.t.\;1\;\leq \;u\;\leq \;\frac{h\times w}{4},$$ (8)

where *I*_*t*_ is the tamper detection result and its definite position (*x*,*y*) is determined by (9).

$$\left\{\begin{array}{c} x=2\times \lfloor \frac{u-1}{w/2}\rfloor +t\\ y=2\times \left((u-1)mod\frac{w}{2}\right)+t \end{array}\right.$$ (9)

where the symbol ⌊·⌋ denotes the least integer function, the parameters *x* and *y* separately range from 1 to *h* and 1 to *w*. Besides, the variable *t* is opted from the number set {1,2}.

Hereto, the visualization image for the first tamper detection result is constructed as *I*_*t*_. To further reduce the false detection rate, some morphological operations that are regarded as the second optimization of tamper detection are applied, including: 1) erode the graphical result; 2) fill the fully enclosed holes; 3) eliminate the marginal holes. Therefore, the ultimate image *I*_*t*_ labeling all tampered regions can be obtained.

#### 2) Image content self-recovery

To recover the potential tampered image $I^{\prime }$, a puzzle *P* and the table of TDL, which is established by the above image authentication process, are applied in the next implementation. It is mentioned above that the watermark information concealed in the tampered regions is irreversibly damaged under malicious tampering with image content. The damage of SPIHT encoding coefficients will impact the visual quality of recovered image and the capability of decoding. Therefore, we propose the RMBM algorithm combined with the SPIHT encoding algorithm to break the independence of image blocks and retrieve the lost recovery data caused by tampering. Besides, the self-recovery location (SRL) and mapped-recovery location (MRL) tables are devised to separately store the recovery data of corresponding image block, and the recovery location (RL) that is convenient for the construction of *I*_*r*_ is also constructed. The definite steps are presented as follows.

***Input*:** Received image $I^{\prime }$ sized *h* × *w*, the selected 8 × 8 puzzle *P* and corresponding TDL ranging from 1 to *h* × *w/4*.

***Output*:** Recovered image *I*_*r*_.

#### Steps of the image content recovery

Step 1: (*Pre-processing*)

Divide all pixels of $I^{\prime }$ into the non-overlapping image block sequence *B*_*u*_ with 2 × 2 pixels, where *u* ∈{1,2,⋯,*h* × *w*/4}. Subsequently, establish and initialize the sizes of SRL, MRL and RL tables to *h* × *w/4*,Step 2: (*Tables construction*)

Retrieve image block *B*_*u*_ in order and execute ***Algorithm II*** to extract its self-recovery bits *R*_*s*_ and mapped-recovery bits *R*_*m*_. Here, *R*_*s*_ and *R*_*m*_ denote the recovery data of image block *B*_*u*_ and *B*_*IHAT*(*u*)_, which are further stored in the corresponding units of SRL and MRL tables, respectively. Then, judge the value of *TDL*(*u*) to construct RL table. Concretely, maintain the original image block when *TDL*(*u*) = 0; in other cases, modify this tampered region according to the preceding constructed MRL table. The specific implementation can be defined as (10).


$$\left\{\begin{array}{l} RL(u)=SRL(u),\\ RL(u)=MRL(HAT(u)), \end{array}\right.\;\;\;\;\;\;\begin{array}{c} TDL(u)=0\\ TDL(u)=1 \end{array}$$ (10)

Step 3: (*Loop judgement*)

If all image blocks in $I^{\prime }$ have been traversed, this step is end, and continues to the next step; otherwise, set *u* = *u* + 1, and then carry out Step 2 repeatedly.

Step 4: (*Self-recovery*)

Convert the contents of RL table to a bitstream, and thus obtain the recovery data *D*_*r*_. The decoded image can be generated by applying the SPIHT decoding algorithm to *D*_*r*_. To improve the visual quality of recovered image *I*_*r*_, we regard $I^{\prime }$ as cover image and then replace only the tampered regions of $I^{\prime }$ with the corresponding image information in decoded image. Hereto, the recovered image *I*_*r*_ with a desirable perceptual quality is constructed.

## VI. Experimental results and discussions

In this section, we conduct numerous experiments to demonstrate the superiority of our proposed scheme, including the performance of watermarked images, tamper detection accuracy, and self-recovery capability. Here, grayscale images sized 512 × 512 in database are applied for experiments, that contains the meaningful tampered images not efficiently discovered with human eyes and further used for comparison with other state-of-the-art works. All experiments are implemented on the MATLAB R2018a programming.

### A. Evaluation metrics

In the following experiments, on the one hand, to theoretically evaluate the accuracy of tamper detection, four metrics defined as (11) to (14) are applied, viz., the *Precision*, *Recall*, false detection ratio (FDR) and false alarm ratio (FAR). Mathematically, the *Precision* and *Recall* can be calculated by the following (11) and (12).


$$Precision\;=\frac{TP}{TP+FP},$$ (11)

$$Recall\;=\frac{TP}{TP+FN},$$ (12)

Here, the sum of correctly detected tampered image pixels is denoted as TP (true positive), while FP (false positive) signifies the number of intact pixels that are falsely alarmed as invalid, and FN (false negative) signifies the number of tampered pixels that are falsely detected as valid. Thus, the Recall is also represents the true detection ratio (TDR), and the definitions of FDR and FAR are given in (13) and (14).

$$FDR\;=\frac{FN}{TP+FN},$$ (13)

$$FAR\;=\frac{FP}{FP+TN},$$ (14)

where TN (true negative) denotes the number of untampered pixels that are correctly detected. Clearly, the tamper detection performance of image authentication technique is much more accurate when the *Precision* and *Recall* representing detection success rate are nearly to 100%, and the FDR and FAR representing detection failure rates are nearly to 0%.

On the other hand, to access our work’s performance in terms of watermarked images and recovered images, three metrics of visual quality are defined. The peak signal-to-noise ratio (PSNR) calculated by (15) is determined as the primary criterion.


$$PSNR=10\times log_{10}\frac{255^{2}}{MSE}(dB)$$ (15)

MSE denotes the mean square error between two images under comparison as described in (16), where *I*(*x*,*y*) and $I^{\prime }(x,y)$ represent the pixel values of these two images.


$$MSE=\frac{1}{h\times w}\times \sum_{x=1}^{h}\sum_{y=1}^{w}{\left(I(x,y)-I^{\prime }(x,y)\right)}^{2}$$ (16)

Except for PSNR, the structural similarity (SSIM) and quality index (QI), defined in (17) and (18), are regarded as another two metrics to evaluate the image visual quality.


$$SSIM=\frac{\left(2\mu_{i}\mu_{j}+c_{1}\right)\left(2\sigma_{ij}+c_{2}\right)}{\left(\mu_{i}^{2}+\mu_{j}^{2}+c_{1}\right)\left(\sigma_{i}^{2}+\sigma_{j}^{2}+c_{2}\right)},$$ (17)

$$QI=\frac{4\sigma_{ij}\mu_{i}\mu_{j}}{\left({\sigma_{i}}^{2}+{\sigma_{j}}^{2}\right)\left({\mu_{i}}^{2}+{\mu_{j}}^{2}\right)},$$ (18)

Here, the variable *σ*_*ij*_ is the covariance between two image blocks *i* and *j* under comparison, and the variables *μ*_*i*_, *μ*_*j*_ and $\sigma_{i}^{2},\sigma_{j}^{2}$ are the mean value and variance of these, respectively. Furthermore, two parameters *c*_1_ and *c*_2_ are calculated by *c*_1_ = (*t*_1_*R*)^2^ and *c*_2_ = (*t*_2_*R*)^2^, in which *t*_1_ = 0.01, *t*_2_ = 0.03 and *R* is the range of image grayscale pixel value. It is intuitively observed that when SSIM and QI converge on 1, these two images are almost identical.

### B. Simulation results

**Fig 7:**
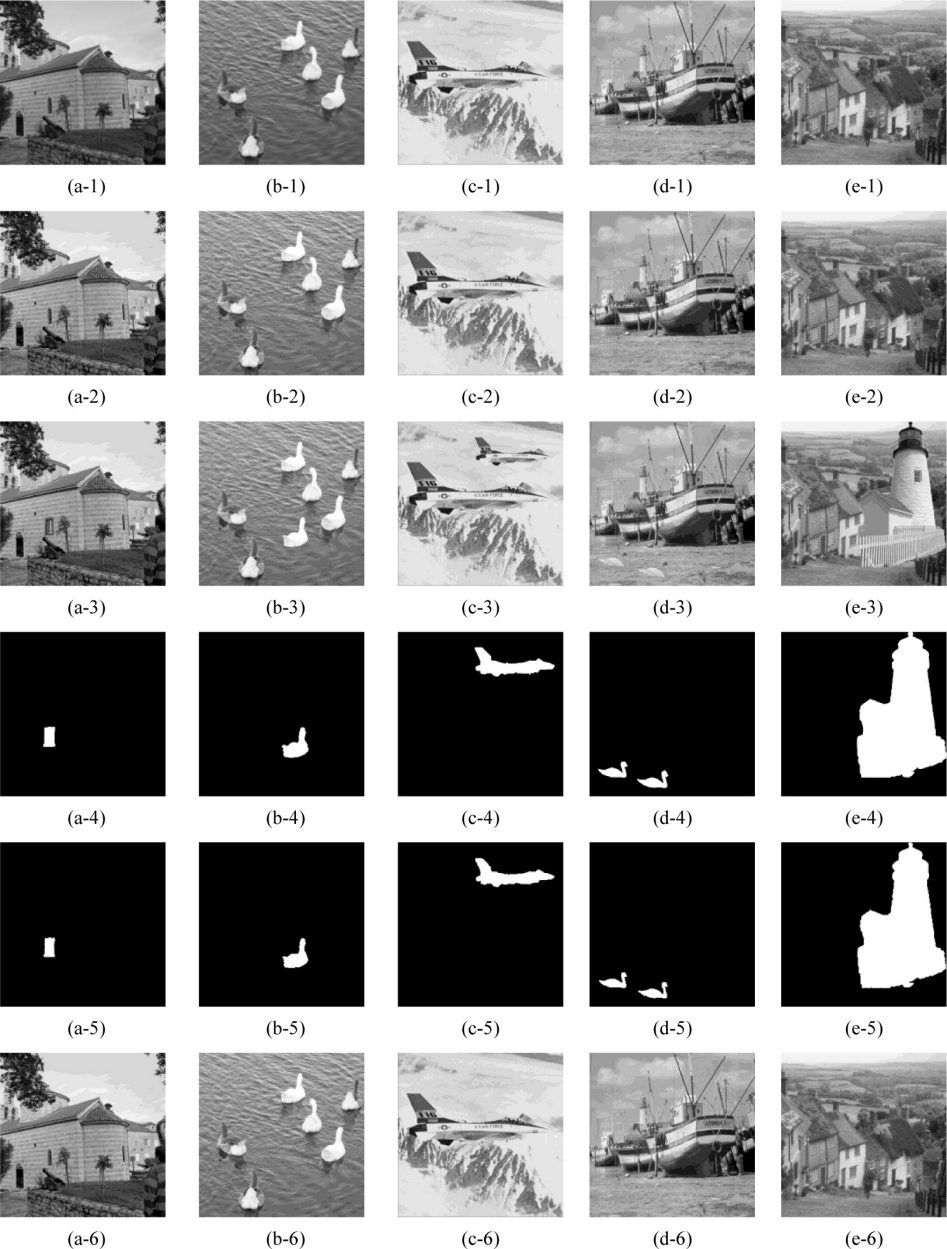
Tamper detection and self-recovery simulation results with the proposed scheme. (a-1)∼(e-1) the original images; (a-2)∼(e-2) the corresponding watermarked images; (a-3)∼(e-3) the tampered images; (a-4)∼(e-4) the ground truth; (a-5)∼(e-5) the tamper detection results; (a-6)∼(e-6) the corresponding recovered images. *The five test images ‘cottage’, ‘swan’, ‘airplane’, ‘boat’, ‘goldhill’ in Fig 7 of our manuscript corresponds to the ‘cottage.tiff’(https://github.com/yooStella/GrayImageDatabase/blob/main/cottage.tiff), ‘swan.tiff’(https://github.com/yooStella/GrayImageDatabase/blob/main/swan.tiff), ‘airplane.tiff’(https://github.com/yooStella/GrayImageDatabase/blob/main/airplane.tiff), ‘boat.tiff’(https://github.com/yooStella/GrayImageDatabase/blob/main/boat.tiff), ‘goldhill.tiff’(https://github.com/yooStella/GrayImageDatabase/blob/main/goldhill.tiff) pictures labelled in this GitHub page.

The tamper detection and recovery results of our proposed scheme are shown in Fig 7, where five test images: ‘cottage’, ‘swan’, ‘airplane’, ‘boat’, and ‘goldhill’ are modified with diverse texture, including smooth, coarse, and edge. The original grayscale images and the corresponding watermarked images that constructed by our proposed scheme are exhibited in the 1^st^ and 2^nd^ rows, respectively. According to the simulation results in 2^nd^ row, we can conclude that after embedding the devised authentication data and recovery data into original images with the DMDH algorithm, the formed watermarked images can still maintain a superior imperceptibility. The 3^rd^ and 4^th^ rows separately list the various tampered images and the ground truth, whose tampering rate are 0.76%, 1.50%, 3.61%, 1.74%, 28.86%. Correspondingly, the 5^th^ row presents the tamper detection results of the 3^rd^ row images, viz., {*Precision*, *Recall*, FDR, FAR} = {100%, 90.10%, 9.90%, 0%}, {99.89%, 90.53%, 9.47%, 0.002%}, {99.82%, 94.18%, 5.82%, 0.011%}, {99.67%, 86.54%, 13.46%, 0.005%}, {100%, 97.12%, 2.88%, 0%}. The 6^th^ row shows the corresponding recovered images with our proposed scheme, viz., {PSNR, SSIM, QI} = {44.05dB, 0.9987, 0.9997}, {43.32dB, 0.9961, 0.9990}, {42.28dB, 0.9909, 0.9992}, {40.29dB, 0.9879, 0.9986}, {33.92dB, 0.9643, 0.9946}. It is intuitive that the proposed block mapping and dual-matrix-based digital watermarking scheme demonstrates excellent performance in tampered region detection and image content self-recovery regardless of the regular or irregular malicious modification. Furthermore, it is also effective for the multiple tampered regions as shown in (d-3). In particular, for images that have been maliciously tampered to be incapable to recognize the original content, as shown in (e-3) with 28.86% tampering rate, our proposed scheme can still recover them to a good perceptual quality.

### C. Analysis on watermarked images

Generally, the performance of watermarked images can be evaluated by quality, watermark payload, and security [34].

#### 1) Visual quality versus watermark payload

To measure the watermark payload of a single image pixel, the embedding rate (ER) is defined as (19).

$$ER=\frac{∥S∥}{h\times w}(bpp),$$ (19)

where ||*S*|| is a statistical value signifying the sum of binary bits concealed in the original image, which is also called embedding capacity (EC). Nevertheless, the metrics of visual quality and watermark payload are contradictory and mutually restricted; in other words, a large payload data hiding method tends to result in a watermarked image of relatively poor visual quality. Table 2 displays the objective results of the DMDH algorithm used in our proposed scheme.

**Table 2:** Experimental results of watermarked images.

Images	EC (bits)	SSIM	QI	ER (bpp)	PSNR (dB)
‘lena’	786,432	0.9915	0.9988	3	40.72
‘peppers’	786,432	0.9912	0.9989	3	40.74
‘camera’	786,432	0.9879	0.9993	3	40.71
‘elaine’	786,432	0.9924	0.9988	3	40.73
‘airplane’	786,432	0.9900	0.9988	3	40.71
‘barbara’	786,432	0.9944	0.9991	3	40.75
‘boat’	786,432	0.9936	0.9988	3	40.75
‘goldhill’	786,432	0.9944	0.9989	3	40.72
Average	786,432	0.9919	0.9989	3	40.73

To specifically demonstrate the merits of DMDH algorithm, a comparison on the watermarked image with aforementioned authentication schemes [23, 24, 27] is presented in Table 3. Intuitively, our proposed scheme has a respective large embedding payload increment of 1 bpp, 1.5 bpp compared to [23, 24]; thus, it is reasonable that the improvement in ER is at the acceptable sacrifice of watermarked image visual quality. Furthermore, the scheme in [27] has the same ER but its value of PSNR is much lower than our proposed scheme.

**Table 3:** Comparison on visual quality and watermark payload.

Images	Haghighi et al. scheme [23]		Prasad et al. scheme [24]		Sreenivas et al. scheme [27]		The proposed scheme	
	ER (bpp)	PSNR (dB)	ER (bpp)	PSNR (dB)	ER (bpp)	PSNR (dB)	ER (bpp)	PSNR (dB)
‘lena’	2	45.82	1.5	42.01	3	37.98	3	40.72
‘peppers’	2	45.80	1.5	42.21	3	37.93	3	40.74
‘camera’	2	45.81	1.5	41.82	3	37.89	3	40.71
‘elaine’	2	45.78	1.5	42.29	3	37.92	3	40.73
‘airplane’	2	45.81	1.5	42.95	3	38.06	3	40.71
‘barbara’	2	45.81	1.5	42.29	3	37.93	3	40.75
‘boat’	2	45.76	1.5	41.11	3	37.98	3	40.75
‘goldhill’	2	45.23	1.5	42.31	3	37.95	3	40.72
Average	2	45.73	1.5	42.12	3	37.96	3	40.73

**Fig 8:**
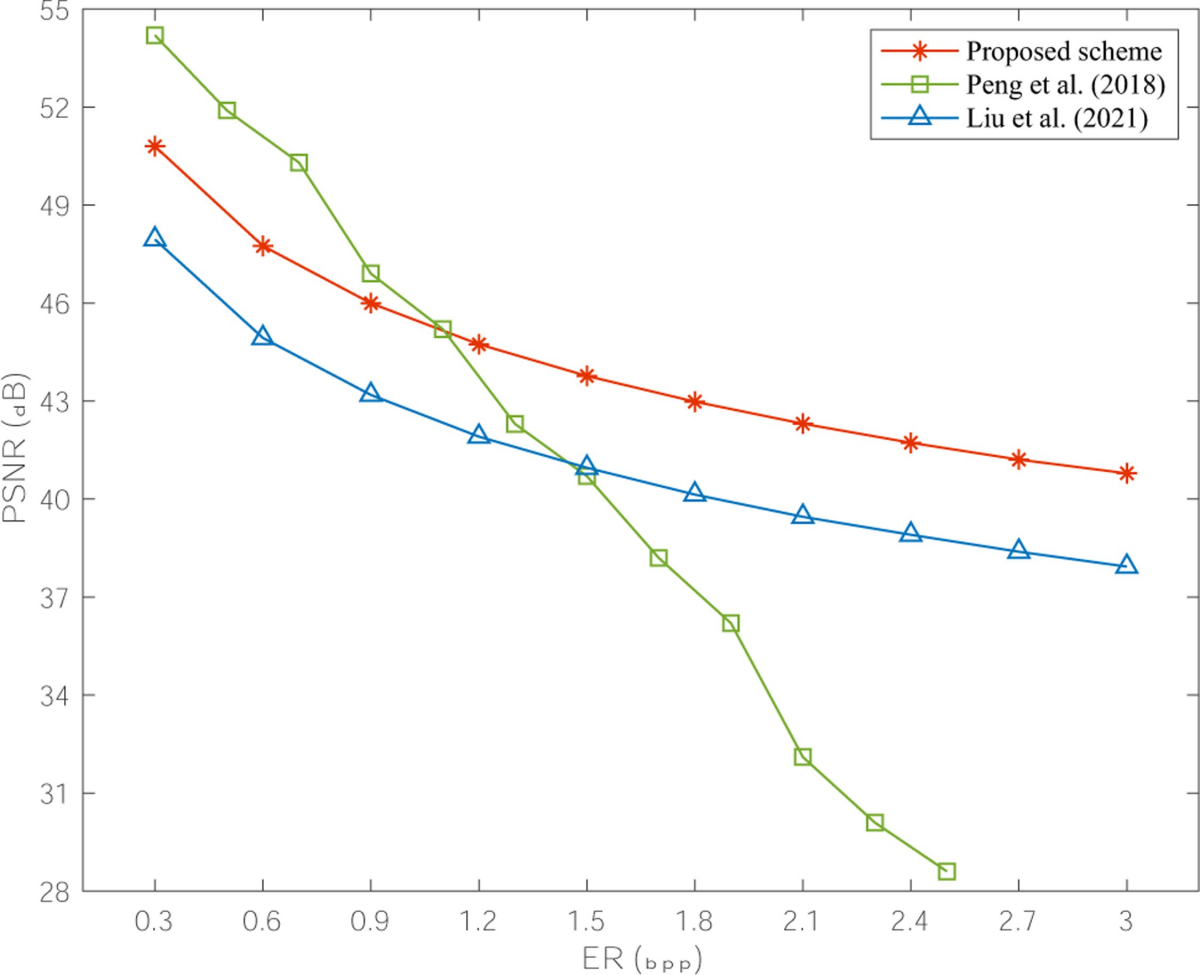
PSNR comparison results under diverse watermark payload.

Fig 8 shows the variation tendency in PSNR average values for the watermarked images obtained by our proposed scheme and two adaptive authentication schemes [12, 20] under the condition of various watermark payload. Intuitively, the curve of our proposed scheme is higher than that of [20], which signifies a smaller image distortion overall. Although the PSNR value of watermarked image yielded by scheme [12] is larger than our proposed scheme when less than 1bpp, this low watermark payload cannot satisfy the practical tampering detection demand. Moreover, to obtain the satisfactory image authentication performance, more embedded data are actually applied in [12], which means a relatively larger image quality loss result as shown in Fig 8.

#### 2) Security analysis

**Fig 9:**
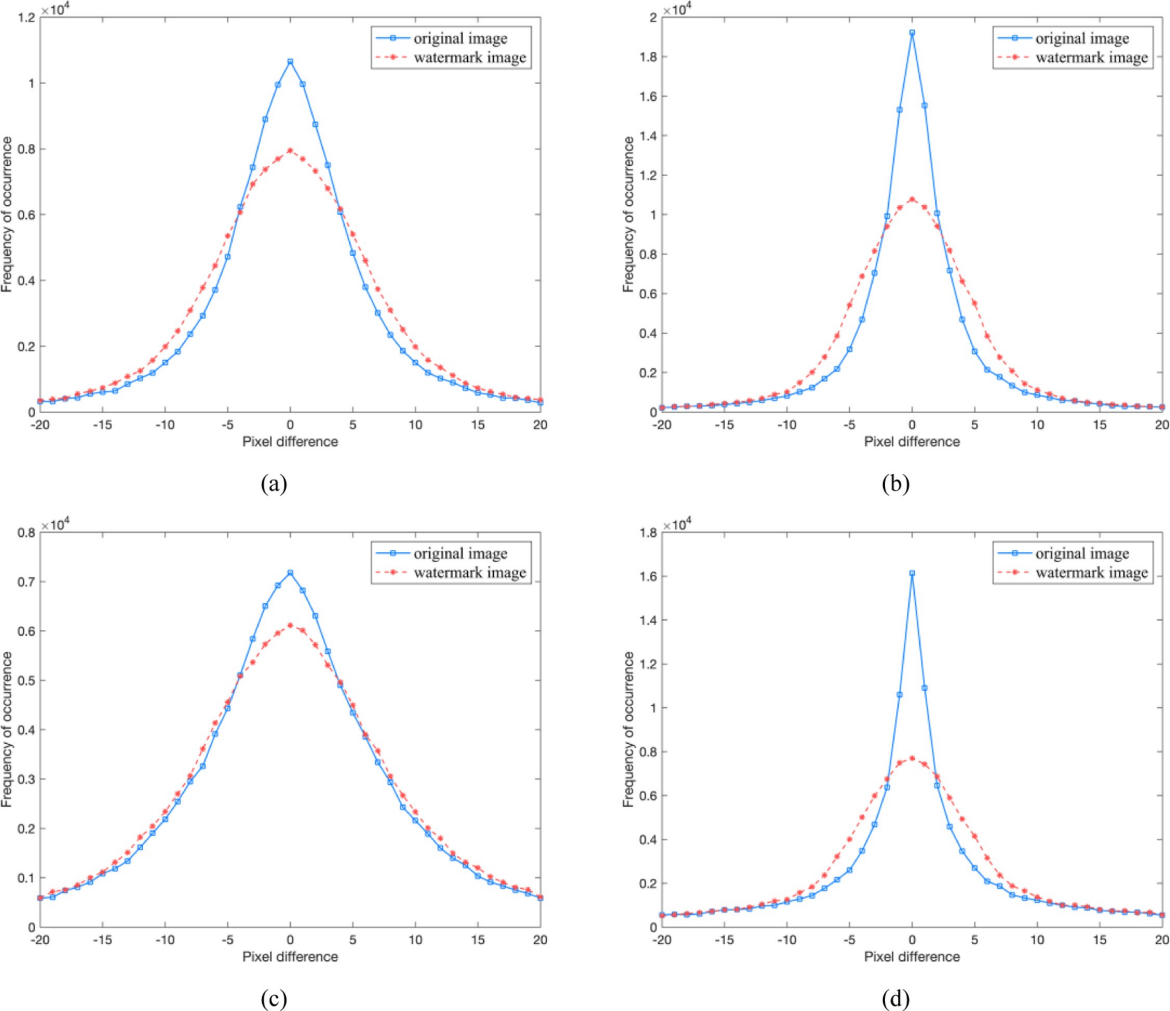
PDH histograms between the original images and watermarked images. (a) Lena; (b) Airplane; (c) Boat; (d) Barbara.

The security of watermarked images can be generally evaluated by the statistical attack and visual attack [34]. In this paper, the pixel-value difference histogram (PDH) analysis and regular/singular (RS) steganalysis [21] are applied to theoretically measure the security of watermarked images, while the enhancing LSBs attack [35] is applied to analyze the visual security. The PDH statistical attack can effectively detect the modification of pixel value differencing. It presents the frequency distribution of the difference values between two contiguous pixels in each pairwise image block, and the horizontal-axis and vertical-axis of the corresponding PDH histogram signify the pixel difference and frequency of it, respectively. Undoubtedly, the PDH curve of original image is macroscopically smooth, viz., no step-effects or zig-zag appearance. As depicted in Fig 9, this characteristic is similar to that of the watermarked image constructed by DMDH algorithm, which means its resistance to PDH statistical attack.

**Fig 10:**
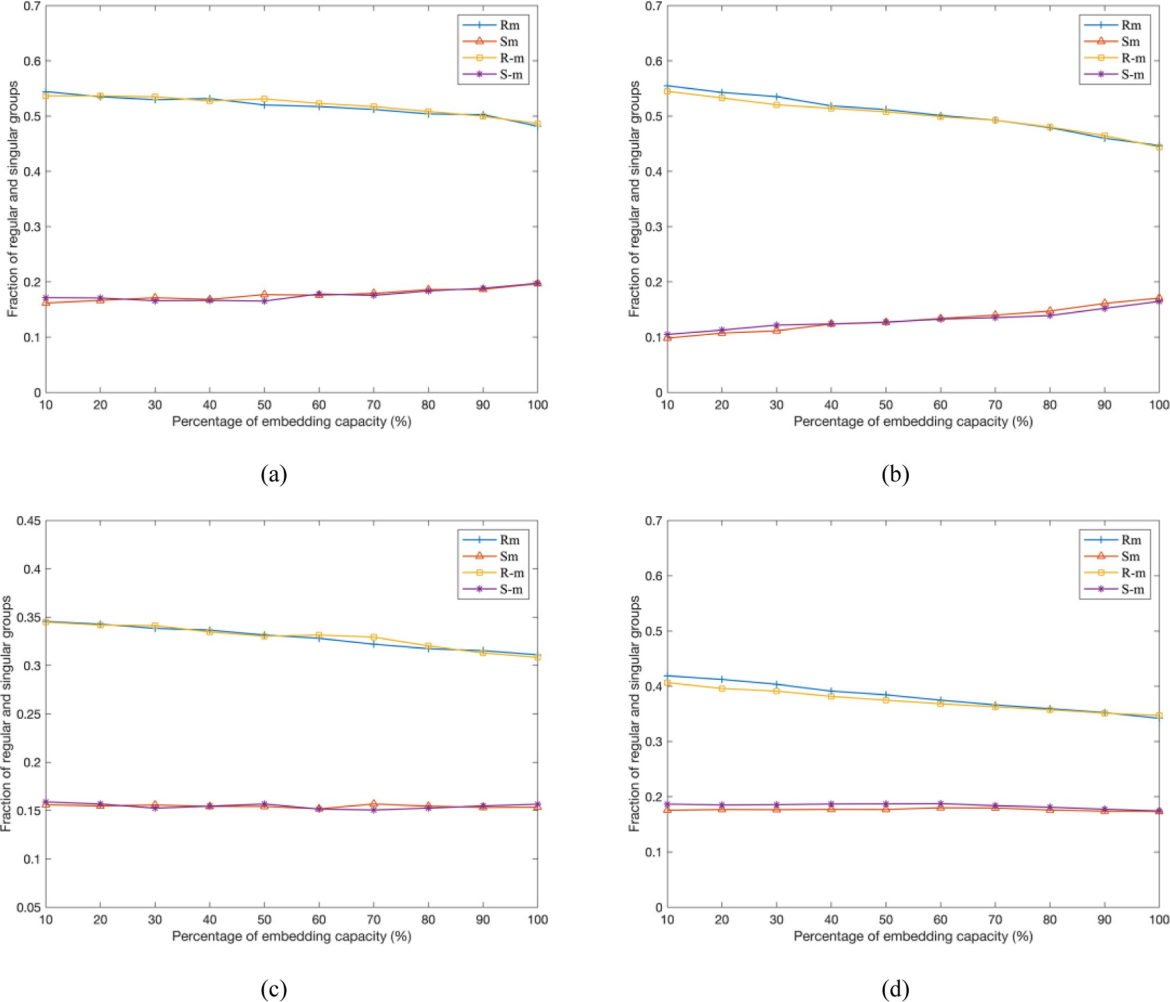
RS diagrams of watermarked images constructed by our proposed scheme. (a) Lena; (b) Airplane; (c) Boat; (d) Barbara.

Except for PDH statistical attack, the other RS steganalysis [21] is also a dual statistical analysis strategy that can exactly detect the LSB embedding operation. It divides the image into non-overlapping image blocks and then classifies these into the unusable group, regular group or singular group according to mask *M*, flipping function *F* and discrimination function *f*. Therefore, the percentages of the regular groups and singular groups are referred to as *R*_*M*_ and *S*_*M*_ for mask *M* while as *R*_-M_ and *S*_-*M*_ for mask -*M*, respectively. Note that for any original image, it satisfies the feature that *R*_*M*_ ≅ *R*_-*M*_ and *S*_*M*_ ≅ *S*_-*M*_. Fig 10 exhibits the RS steganalysis diagrams of watermarked images where the ordinate denotes the percentage of regular and singular groups with mask *M* and -*M*, and the abscissa denotes the percentage of embedding capacity. It is intuitively deduced that the DMDH algorithm employed in our proposed scheme can resist the image statistical attack, thus it is extremely secure.

**Fig 11:**
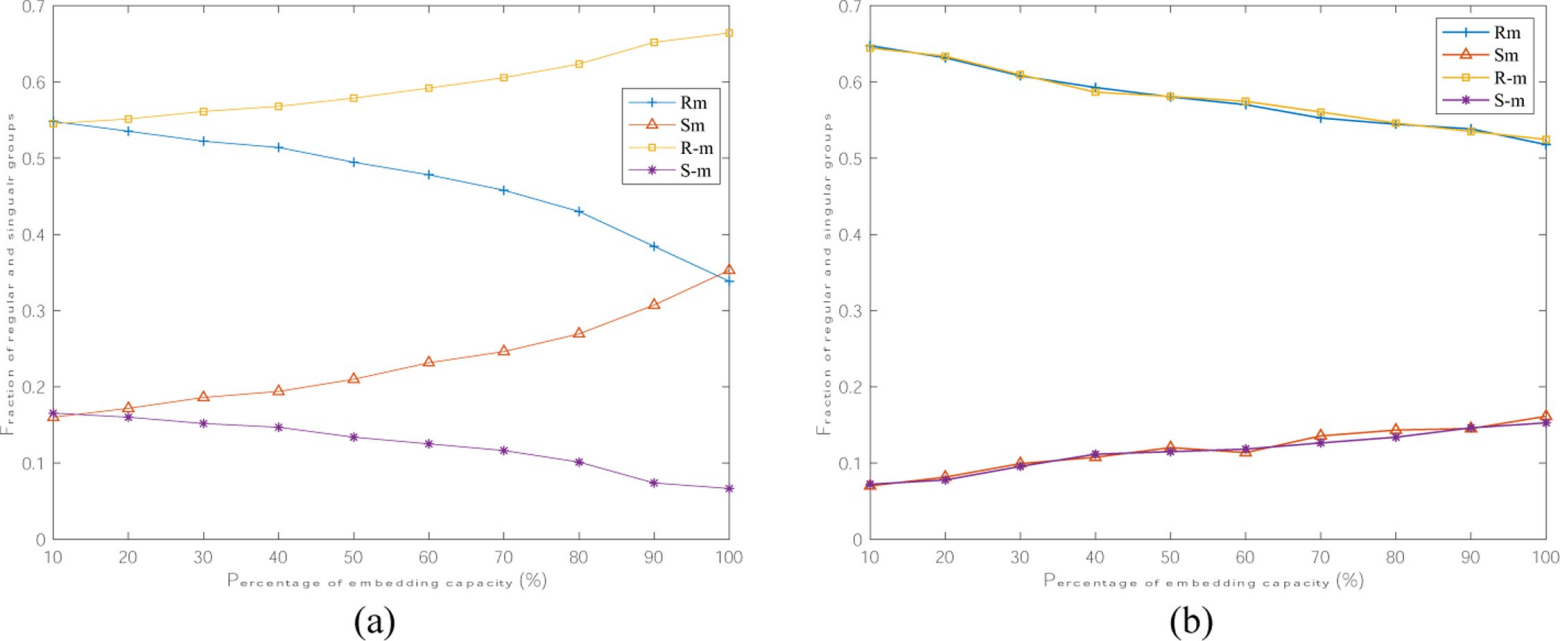
RS-diagram comparison on the image ‘camera’. (a) Liu et al. scheme [20]; (b) the proposed scheme.

The security comparison on watermarked image between the proposed scheme and the latest scheme [20] is shown in Fig 11. Obviously, with the increase of watermark payload, the curves of regular or singular groups for mask *M* and -*M* are become more and more separated in (a), while the expected values of *R*_*M*_ and *S*_*M*_ are approximately identical with that of *R*_-M_ and *S*_-*M*_ in (b). Therefore, the RS diagram of ‘camera’ image with the proposed scheme is extremely closer compared to [20].

**Fig 12:**
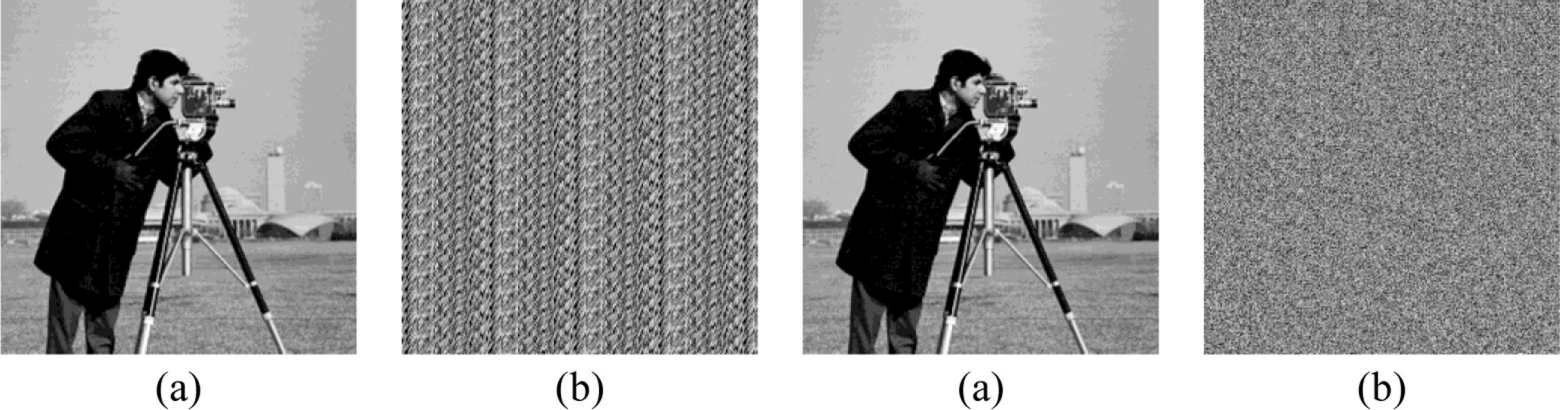
Enhancing LSBs visual attack (*k* = 3) on the image ‘camera’. (a) watermarked image with Liu et al. scheme [20]; (b) attack on image (a); (c) watermarked image with the proposed scheme; (d) attack on image (c). * The image ‘camera’ in Fig 12 of manuscript corresponds to the ‘cameraman.tiff’(https://github.com/yooStella/GrayImageDatabase/blob/main/cameraman.tiff) picture labelled in this GitHub page.

The enhancing LSBs visual attack [35] extracts *k* LSBs of each pixel and takes them as the MSBs followed by the 8–*k* sized 0 bits to form a novel image pixel. As shown in Fig 12, the visual attack on (a) generated by [20] will form a certain regular pattern as (b), which reveals the embedding operation. In contrast, the pattern image constructed via the visual attack will appear in chaos as (d), when the watermarked image is generated by our proposed scheme, which can successfully avert suspicions from the malicious attackers. Consequently, we can make a solid statement that the proposed scheme is comparatively more secure than [20].

### D. Analysis on tamper detection and recovery performance

**Fig 13:**
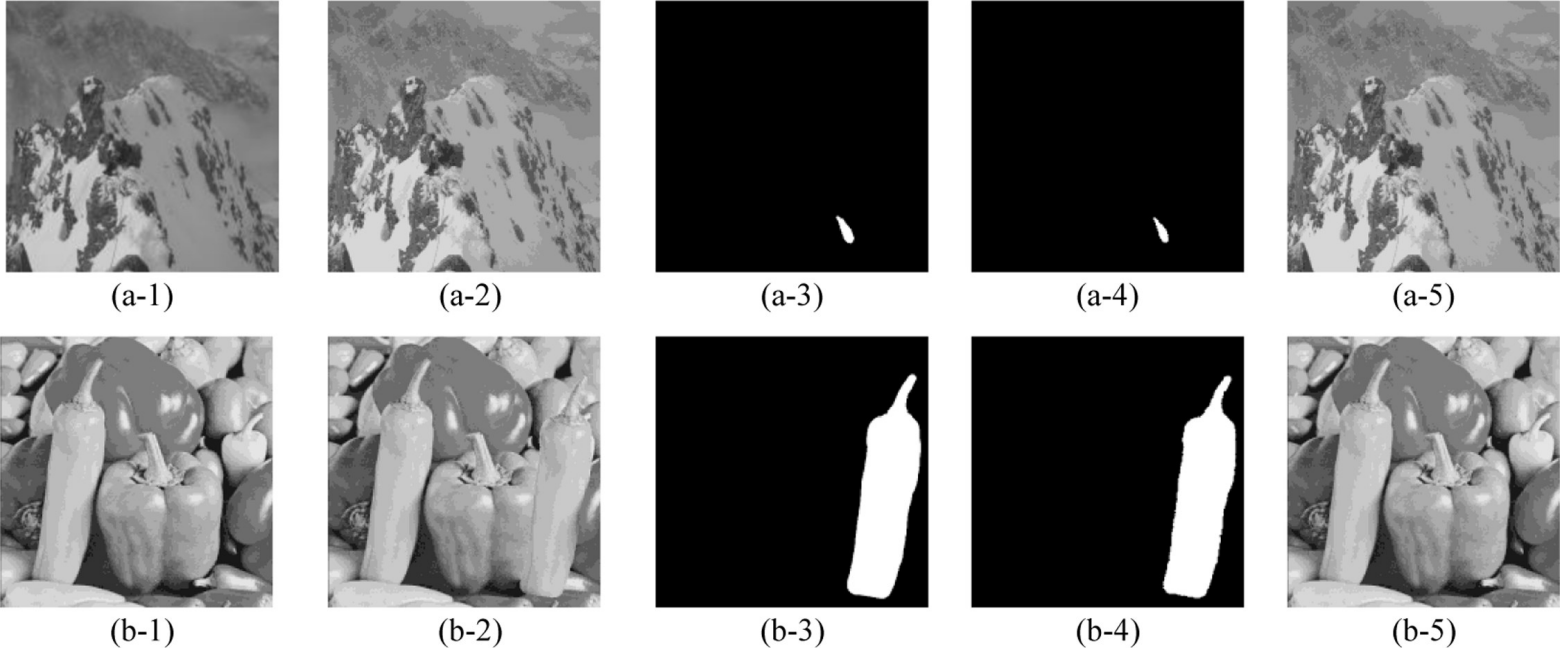
Performance of the proposed scheme under copy-move attack. (a-1), (b-1) the original image; (a-2), (b-2) the corresponding image tampered by copy-move attack; (a-3), (b-3) the ground truth; (a-4), (b-4) the tamper detection result; (a-5), (b-5) the recovered image. *The images ‘snowberg’, ‘peppers’ in Fig 13 of our manuscript corresponds to the ‘snowberg.tiff’(https://github.com/yooStella/GrayImageDatabase/blob/main/snowberg.tiff), ‘peppers.tiff’(https://github.com/yooStella/GrayImageDatabase/blob/main/peppers.tiff) pictures labelled in this GitHub page.

**Fig 14:**
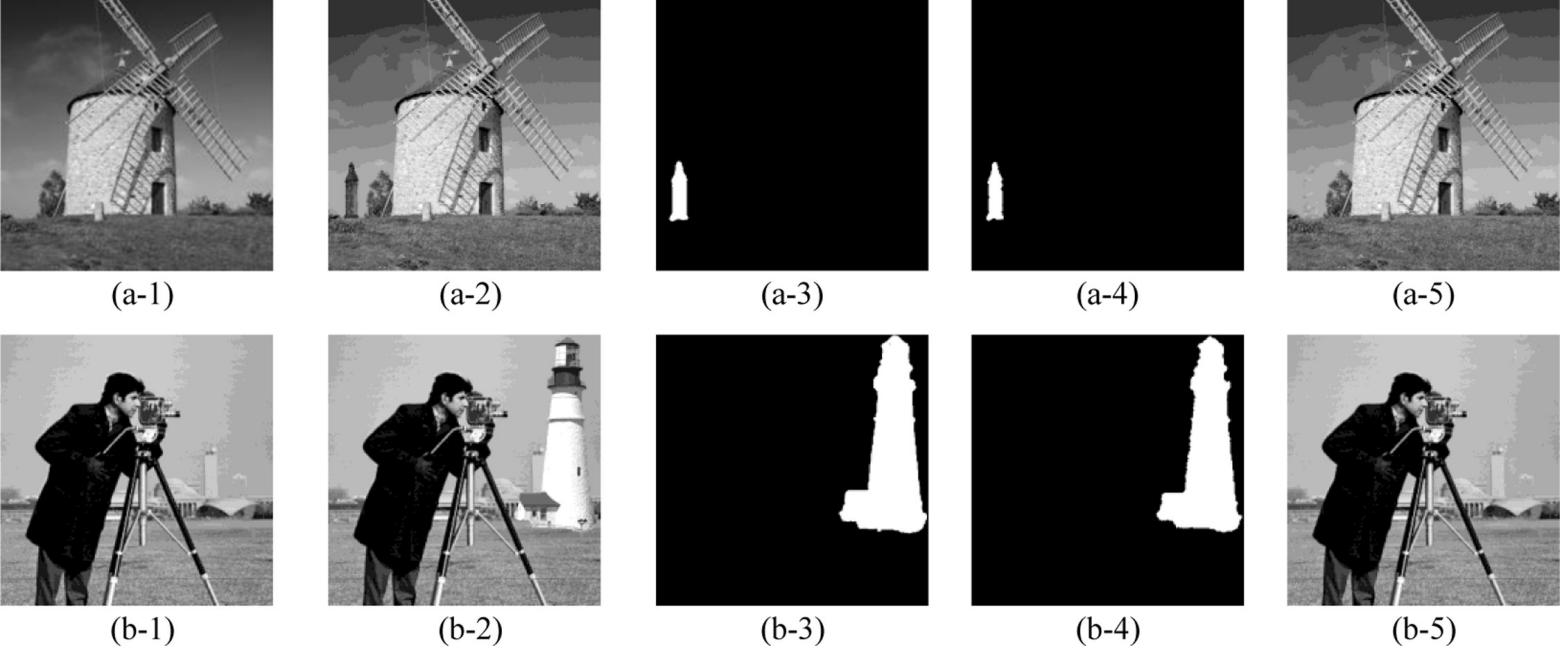
Performance of the proposed scheme under collage attack. (a-1), (b-1) the original image; (a-2), (b-2) the corresponding image tampered by collage attack; (a-3), (b-3) the ground truth; (a-4), (b-4) the tamper detection result; (a-5), (b-5) the recovered image. * The images ‘windmill’, ‘camera’ in Fig 14 of our manuscript corresponds to the ‘windmill.tiff’(https://github.com/yooStella/GrayImageDatabase/blob/main/windmill.tiff), ‘cameraman.tiff’(https://github.com/yooStella/GrayImageDatabase/blob/main/cameraman.tiff) pictures labelled in this GitHub page.

Considering that digital images can be easily tampered with via the Internet transmission, and an effective image integrity authentication technique should detect and locate the tampered regions under diverse malicious attacks. In this subsection, we conduct the common attacks of copy-move attack and collage attack to verify the efficiency of our proposed mechanism. The copy-move attack replaces the part of image with the content of itself from another position. However, some existing works [19, 27] cannot resist the copy-move attack owing to the independency of image blocks, for example, [19] uses the hash function to construct the authentication bits from the image’s MSB. The collage attack uses parts of at least two different image to form a tampered image and retains the same relevant spatial position. Actually, the proposed scheme can effectively resist the copy-move attacks and collage attack as simulation results shown in Figs 13 and 14. Among them, the original images are shown in the 1^st^ column, which are ‘snowberg’, ‘peppers’, ‘windmill’, and ‘camera’. The 2^nd^ column exhibits the corresponding image tampered by copy-move attack and collage attack, respectively. The 3^rd^ column is the ground truth, whose tampering rates are 0.3%, 12.90% in Fig 14, and 1.03%, 12.04% in Fig 14. Correspondingly, the 4^th^ column list the tamper detection result, viz., {Precision, Recall, FDR, FAR} = {100%, 73.91%, 26.09%, 0%}, {99.98%, 97.26%, 2.74%, 0.004%} in Fig 13, {Precision, Recall, FDR, FAR} = {99.92%, 90.96%, 9.04%, 0.001%}, {99.98%, 92.38%, 7.62%, 0.002%} in Fig 14. The 5^th^ column list the recovered image, viz., {PSNR, SSIM, QI} = {61.24dB, 0.9997, 0.9991}, {41.83dB, 0.9936, 0.9991} in Fig 13, {PSNR, SSIM, QI} = {47.30dB, 0.9952, 0.9998}, {42.10dB, 0.9896, 0.9995} in Fig 14.

**Fig 15:**
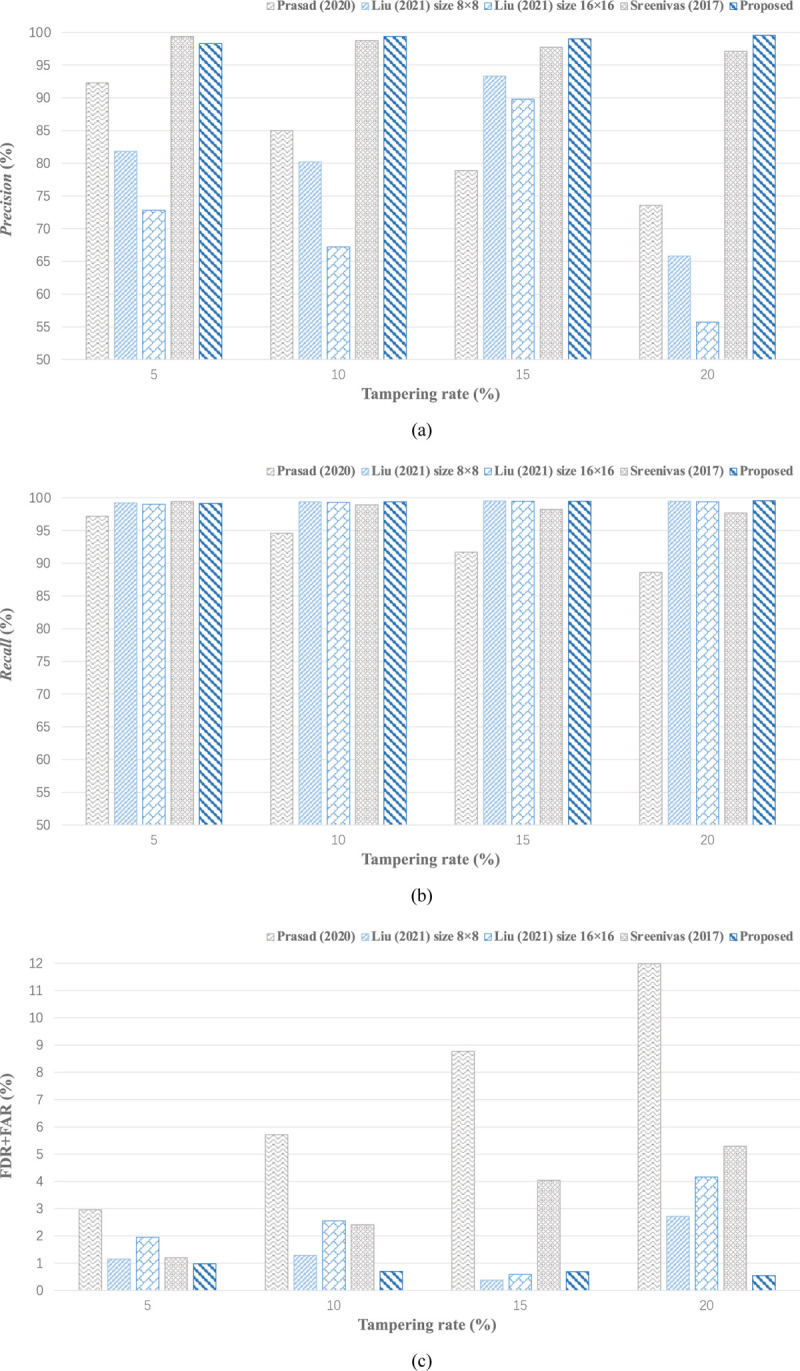
Comparisons on tamper detection results under the diverse tampering rate. (a) *Precision* variation tendency; (b) *Recall* variation tendency; (c) FDR and FAR variation tendency.

In the following part, to evaluate the proposed scheme in a comprehensive way, we conduct the comparison experiments with the state-of-the-art schemes in terms of tamper detection and recovery performance. The accuracy of tamper detection is estimated by *Precision*, *Recall*, FDR, and FAR; and the quality of recovered image is primarily estimated by PSNR. Fig 15 presents, under the various tampering rates, the simulation result comparisons of our proposed scheme and three effective schemes [20, 24, 27]. As is evident from (a), the statistical values of our proposed scheme generally outperform the above schemes with respect to *Precision*; and maintain the relatively slight variance compared to others, especially for [20] where the block size makes a visible impact on it. The image reconstruction is conducted in the recovery procedure, thus a high *Precision* is essential for ensuring the desirable visual quality of recovered images. Similarly, the proposed scheme has the overall outperformance in *Recall* and FDR+FAR as exhibited in (b) and (c).

**Fig 16:**
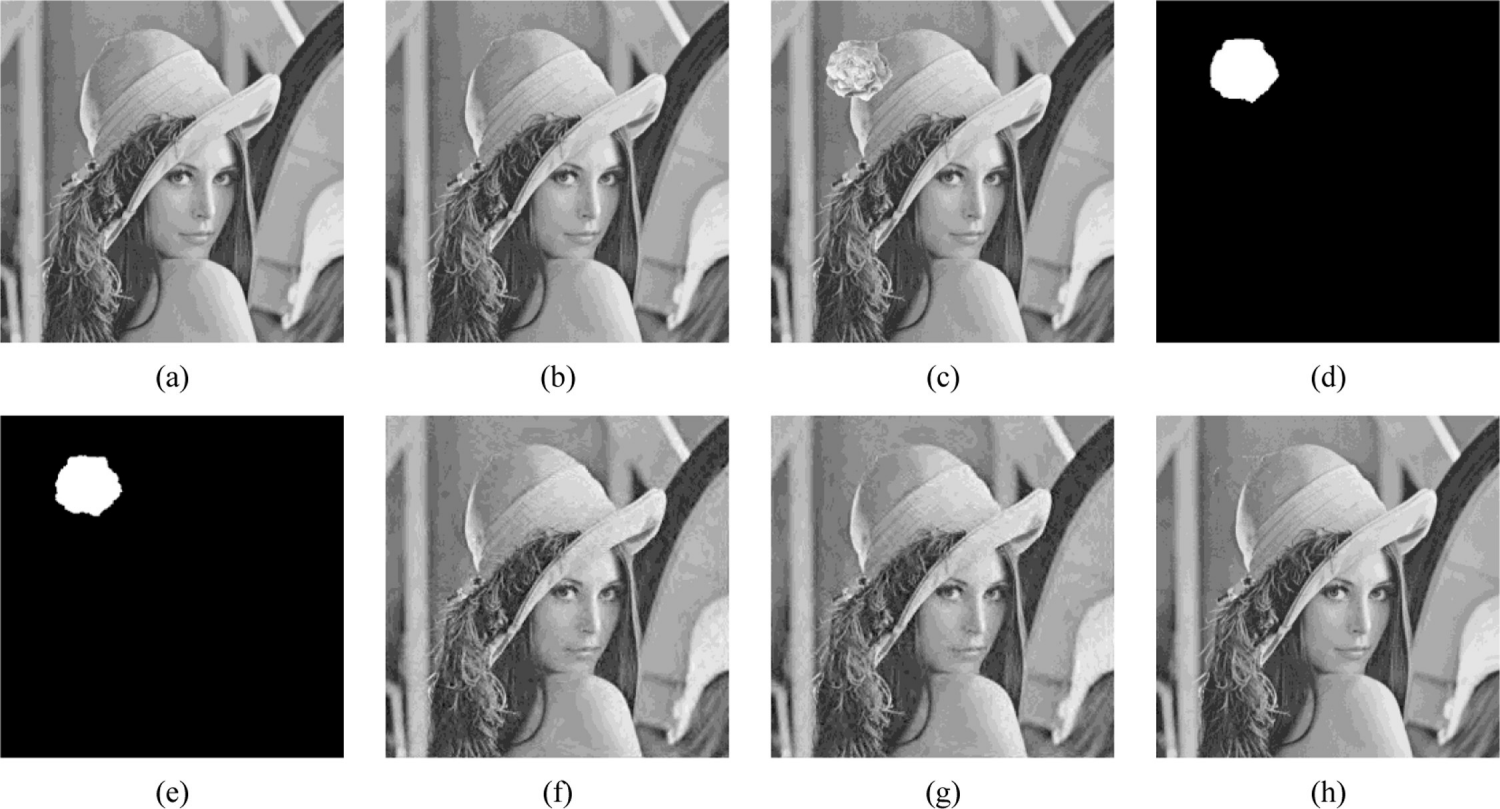
The superiority demonstration of our proposed scheme on image ‘lena’; (a) original image; (b) corresponding watermarked image; (c) tampered image; (d) ground truth, tampering rate: 2.76%; (e) tamper detection result: {*Precision*, *Recall*, FDR, FAR} = {100%, 92.17%, 7.83%, 0%}; (f) recovered image constructed by the baseline model [19] without mapped-recovery bits guarantee: {PSNR, SSIM, QI} = {27.88dB, 0.8435, 0.9770}; (g) decoded image with the proposed scheme; (h) recovered image constructed by the proposed scheme: {PSNR, SSIM, QI} = {43.33dB, 0.9916, 0.9993}. *The image ‘lena’ in Fig 16 of manuscript corresponds to the ‘lena.tiff’(https://github.com/yooStella/GrayImageDatabase/blob/main/lena.tiff) picture labelled in this GitHub page.

With respect to the recovery performance, to demonstrate the superiority of our work in image recovery procedure, we conduct a visual quality comparison on the recovered image between the proposed scheme and baseline model [19] without mapped-recovery bits guarantee, as exhibited in Fig 16. It is apparent that the loss of recovery data in [19] will inevitably lead to the decrease of recovered image quality as shown in (f). Yet with our proposed RMBM algorithm that guarantees tampered region recovery, the image can be successfully decoded. Consequently, the better visual quality of recovered image can be constructed, shown as the {PSNR, SSIM, QI} comparison result in (h).

**Fig 17:**
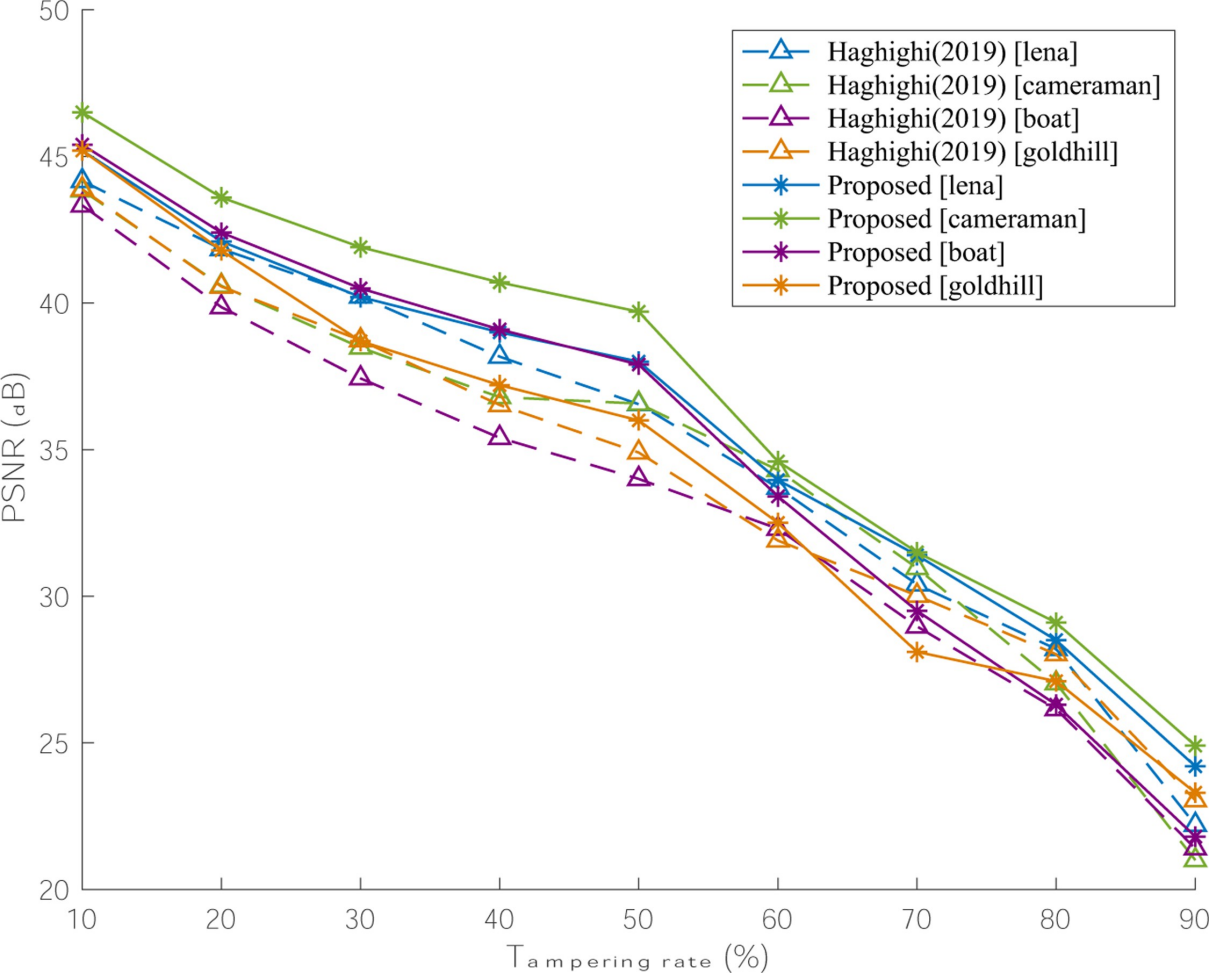
PSNR variation tendency of the recovered images under the diverse tampering rate.

In Fig 17, four representative images of various texture: ‘lena’, ‘camera’, ‘boat’, and ‘goldhill’ are tampered from 10% to 90%, with a step of 10%. Accordingly, the PSNR results of recovered images are separately shown in Fig 17, where the scheme of [23] is given for comparison. It is clearly observed that with the increment of tampering rate, the visual quality of recovered image drops and our proposed scheme can still have a superior performance in tampered region recovery.

In addition, more PSNR comparisons with existing works on recovered images are listed in Table 4. Here, the tampering rate varies from 10% to 50%, with a step of 10%, and the corresponding average PSNR results of six recovered images ‘lena’, ‘cameraman’, ‘boat’, ‘goldhill’, ‘peppers’, ‘airplane’ are listed for comparison. The results of our proposed scheme are highlighted in italic, while the best values are highlighted in bold. Intuitively, our work outperformances these schemes overall with respect to the visual quality of recovered images. According to the above comparisons, it can be concluded that the proposed scheme has the superior performance of tamper detection accuracy, strong capability of image content self- recovery and effective resistance to malicious attack.

**Table 4:** PSNR comparisons with existing works on recovered images.

Schemes		Tampering rate (%)				
		10	20	30	40	50
Haghighi et al. [23]		43.41	40.81	38.94	37.02	35.87
Liu et al. [20]	8×8 size	44.58	41.23	37.86	35.44	33.89
	16×16 size	38.12	35.64	34.09	32.67	31.53
Singh et al. [25]		43.77	41.52	37.31	35.80	34.66
Qin et al. [26]		35.53	33.28	32.07	31.15	-
Sreenivas et al. [27]		40.76	37.25	33.18	30.79	28.58
Dadkhah et al. [36]		43.02	37.92	33.01	32.23	31.14
Proposed		***45*.*28***	***42*.*59***	***40*.*12***	***38*.*97***	***37*.*76***

*: ‐ signifies the recovered image is unavailable for current tampering.

## VII. Conclusions and future works

A novel image authentication technique with self-recovery capability using block mapping and dual-matrix-based fragile watermarking is elaborately reviewed in this research paper. The principal regulation of our proposed scheme is to initially regard non-overlapping 2 × 2 pixels as an image block, and then construct the authentication data for tampered region localization and recovery data for image content self-recovery from the original image. The AFCC algorithm is presented to obtain the authentication bits, including the HIT feature bits generated by the RMBM algorithm and the parity check bits generated by the BDPC algorithm. To break the independence of image blocks and provide a guarantee of recovered image quality, we additionally design the mapped-recovery bits to construct recovery data together with the self-recovery bits. The SPIHT algorithm is used to generate the self-recovery bits, and then combined with the RMBM algorithm to generate the mapped-recovery bits. Ultimately, both the authentication data and recovery data will constitute the watermark information, which is further embedded into the original image through the devised DMDH algorithm.

The experimental analysis demonstrates that our proposed scheme has the merit of tamper detection accuracy and image self-recovery performance compared to other state-of-the-art works; furthermore, it maintains a satisfactory watermarked image quality and the effective attack resistance. Considering that our proposed scheme needs a large watermark payload to improve the tamper detection accuracy, which comes at the sacrifice of watermarked image visual quality. Therefore, the main challenge we will focus on the future work is reducing the watermark payload while maintaining superior detection and self-recovery performance. On the other respect, this proposed scheme is designed for tamper detection and self-recovery mechanism of grayscale images. Accordingly, except for the grayscale images, we will proceed to research the image authentication work for color images.
